# Supplementary material for: Genomic Dissection and Diurnal Expression Analysis Reveal the Essential Roles of the PRR Gene Family in Geographical Adaptation of Soybean
Source: Int J Mol Sci. 2022 Sep 1;23(17):9970. doi: 10.3390/ijms23179970 (PMC9456279; doi:10.3390/ijms23179970)
Supplement: Supplementary file 1 [file ijms-23-09970-s001.zip › ijms-1865446-supplementary.pdf]

Supplementary Materials

This PDF file includes:

Supplementary Figures S1–S4

Supplementary Tables S1–S4

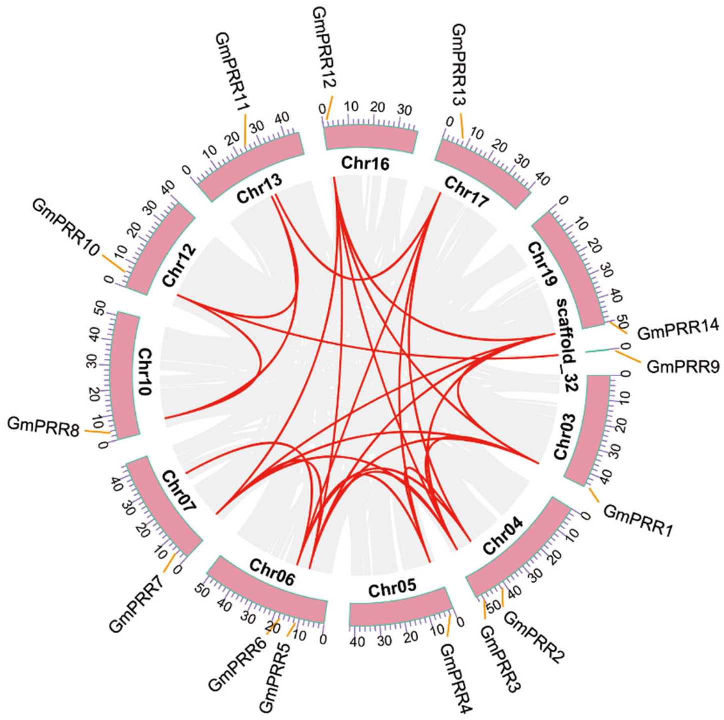

Supplementary Figure S1. Synteny analysis and gene duplication events of *GmPRRs*. Gray lines indicate all synteny blocks in the soybean genome, and the red lines indicate duplicated PRR gene pairs.

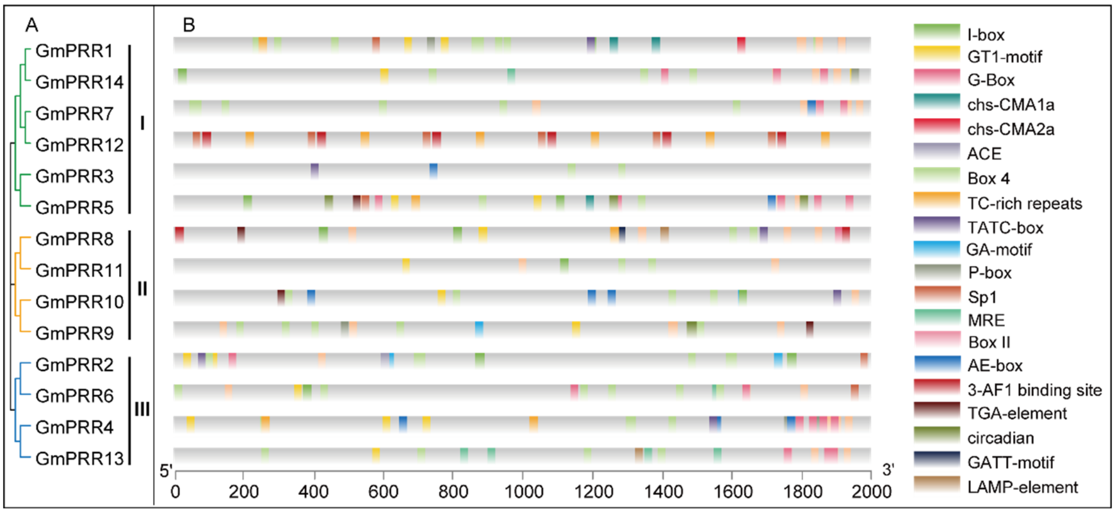

Supplementary Figure S2. Analysis of cis-acting elements in the promoter region of *GmPRRs*. (**A**) Phylogenetic tree of soybean PRR proteins. Details of groups are represented by different colors. (**B**) Types and positions of cis-acting elements in the 2 kb promoter region. The cis-elements are displayed in different color boxes.

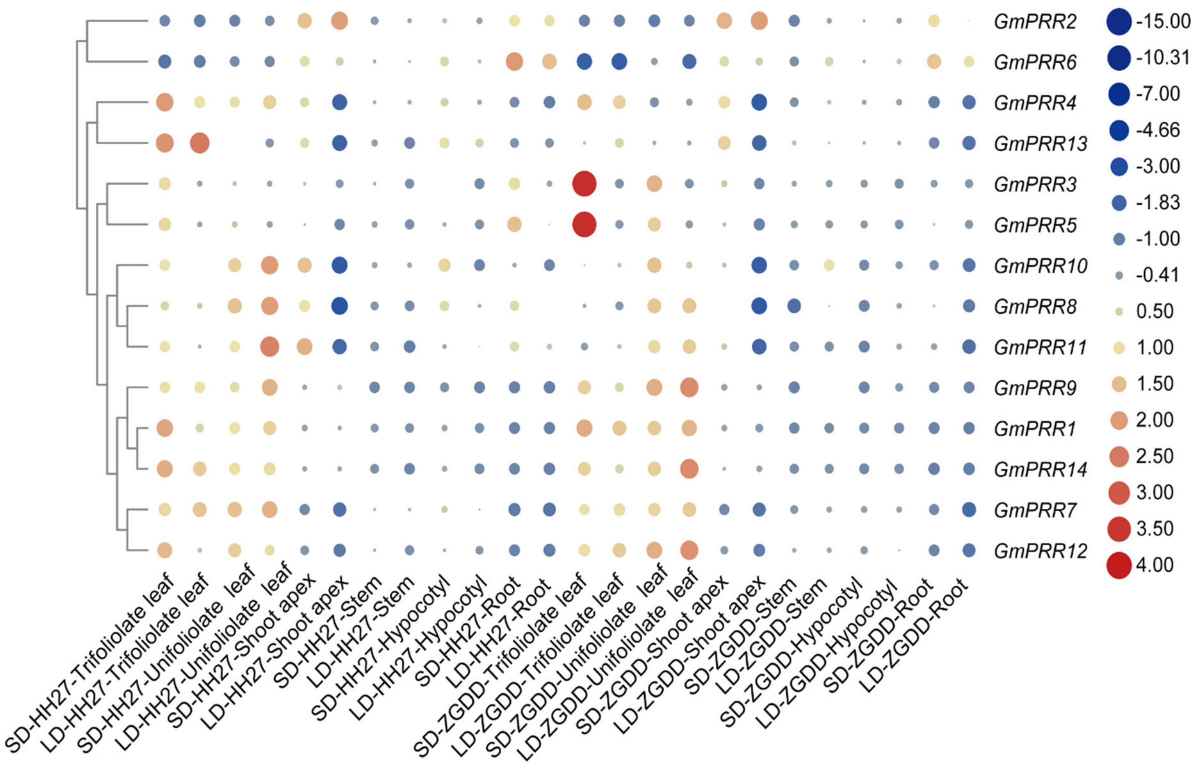

Supplementary Figure S3. Expression profile analysis of *GmPRRs* in different tissues from soybean varieties Zigongdongdou (ZGDD) and Heihe 27 (HH27) under long-day (LD, 16: 8 h, light: dark) and short-day (SD, 12: 12 h, light: dark) treatments. Samples were collected 4 h after exposure to light.

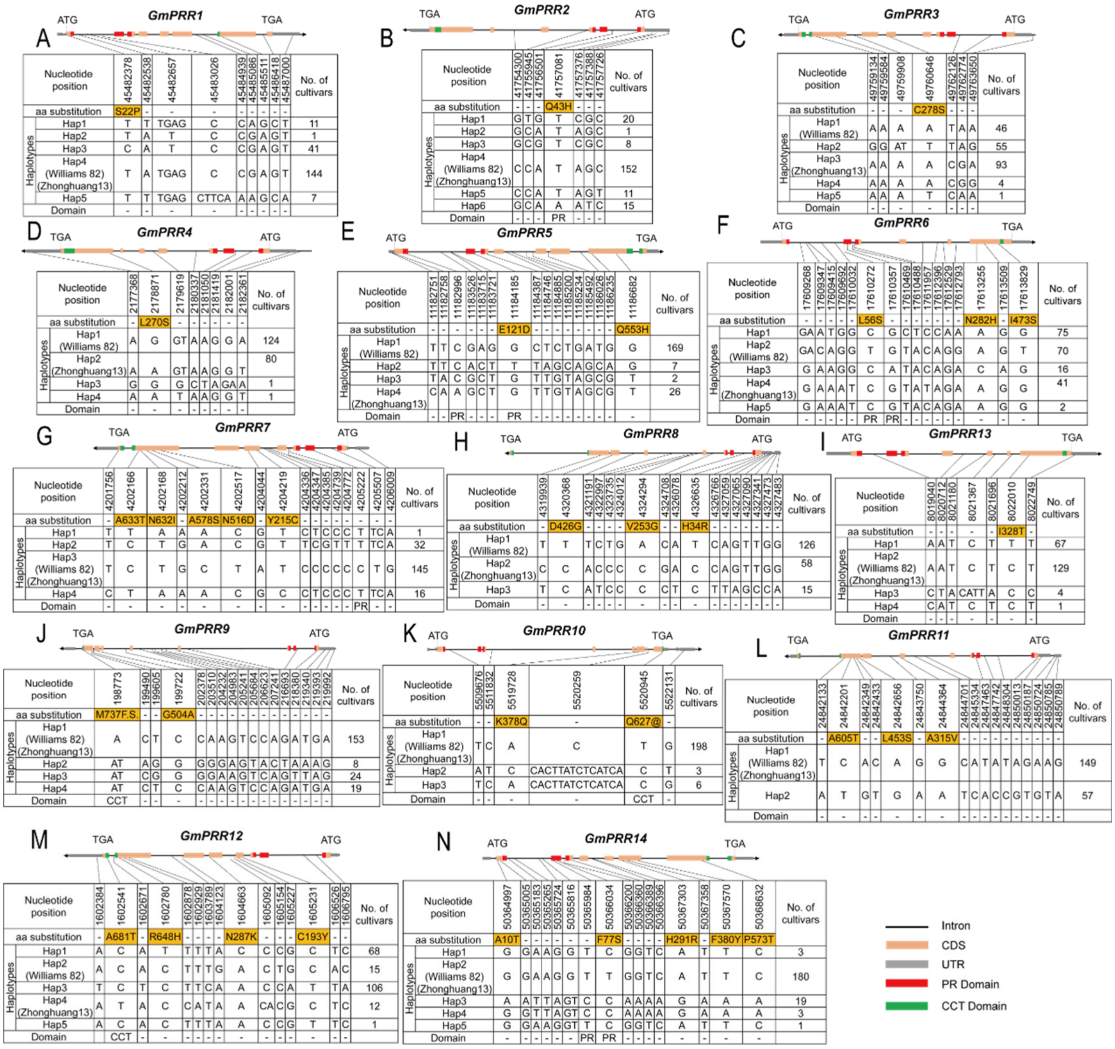

Supplementary Figure S4. Natural variation in nucleotide and amino acid (aa) sequences of *GmPRRs* in the 207 resequencing soybean varieties. Variations that result in protein changes are colored yellow. The aa substitutions are indicated, as well as F.S. (frameshift) and @ (stop codon) changes.

Supplementary Table S1. Physicochemical properties of PRR family proteins in soybean

Gene name	Gene ID	Molecular weight/kDa	isoelectric point	Amino acid number/aa	Predicted Subcellular location
GmPRR1	*Glyma.03G261300*	76.5	6.30	692	Nuclear
GmPRR2	*Glyma.04G166300*	62.6	5.64	560	Nuclear
GmPRR3	*Glyma.04G228300*	76.4	6.43	691	Nuclear
GmPRR4	*Glyma.05G025000*	64.2	5.55	575	Nuclear
GmPRR5	*Glyma.06G136600*	77.1	6.67	700	Nuclear
GmPRR6	*Glyma.06G196200*	62.2	5.56	558	Nuclear
GmPRR7	*Glyma.07G049400*	79.7	5.69	722	Nuclear
GmPRR8	*Glyma.10G048100*	81.8	6.77	753	Nuclear
GmPRR9	*Glyma.U034500*	83.3	5.85	765	Nuclear
GmPRR10	*Glyma.12G073900*	68.4	6.04	626	Nuclear
GmPRR11	*Glyma.13G135900*	81.9	8.01	755	Nuclear
GmPRR12	*Glyma.16G018000*	78.3	5.70	706	Nuclear
GmPRR13	*Glyma.17G102200*	64.7	5.93	579	Nuclear
GmPRR14	*Glyma.19G260400*	76.1	6.63	685	Nuclear

Supplementary Table S2. The information of 207 resequencing soybean varieties

Variety	Region	Ecotype	MG	Source
Beihudou	NE	NSP	MG 00	Heilongjiang, China
Beifeng2	NE	NSP	MG 0	Heilongjiang, China
Beifeng9	NE	NSP	MG 0	Heilongjiang, China
Beifeng11	NE	NSP	MG 0	Heilongjiang, China
Beidou5	NE	NSP	MG 0	Heilongjiang, China
Dengke1	NE	NSP	MG 0	Inner Mongolia, China
Dongnong4	NE	NSP	MG 0	Heilongjiang, China
Dongnong72-806	NE	NSP	MG 0	Heilongjiang, China
Fengshou10	NE	NSP	MG 0	Heilongjiang, China
Fengshou12	NE	NSP	MG 0	Heilongjiang, China
Fengshou17	NE	NSP	MG 0	Heilongjiang, China
Fengshou19	NE	NSP	MG 0	Heilongjiang, China
Fengshou24	NE	NSP	MG 0	Heilongjiang, China
Hefeng25	NE	NSP	MG 0	Heilongjiang, China
Hefeng35	NE	NSP	MG 0	Heilongjiang, China
Heihe3	NE	NSP	MG 0	Heilongjiang, China
Heihe9	NE	NSP	MG 0	Heilongjiang, China
Heihe18	NE	NSP	MG 0	Heilongjiang, China
Heihe19	NE	NSP	MG 0	Heilongjiang, China
Heihe27	NE	NSP	MG 0	Heilongjiang, China
Heihe38	NE	NSP	MG 0	Heilongjiang, China
Heihe51	NE	NSP	MG 0	Heilongjiang, China
Heihe54	NE	NSP	MG 0	Heilongjiang, China
Heilongjiang41	NE	NSP	MG 0	Heilongjiang, China
Huajiang4	NE	NSP	MG 0	Heilongjiang, China
Jinyuan2	NE	NSP	MG 0	Heilongjiang, China
JianMGodou1	NE	NSP	MG 0	Heilongjiang, China
Jingshanpu	NE	NSP	MG 0	Heilongjiang, China
Kexi283	NE	NSP	MG 0	Heilongjiang, China
Kangxian4	NE	NSP	MG 0	Heilongjiang, China
Mancangjin	NE	NSP	MG 0	Heilongjiang, China
Suinong3	NE	NSP	MG 0	Heilongjiang, China
Suinong28	NE	NSP	MG 0	Heilongjiang, China
Suinong41	NE	NSP	MG 0	Heilongjiang, China
Mengdou30	NE	NSP	MG 0	Inner Mongolia, China
Hejiao6	NE	NSP	MG I	Heilongjiang, China
Hejiao8	NE	NSP	MG I	Heilongjiang, China
Hefeng22	NE	NSP	MG I	Heilongjiang, China
Hefeng45	NE	NSP	MG I	Heilongjiang, China
Hefeng47	NE	NSP	MG I	Heilongjiang, China
Hefeng50	NE	NSP	MG I	Heilongjiang, China
Hefeng55	NE	NSP	MG I	Heilongjiang, China
Heinong16	NE	NSP	MG I	Heilongjiang, China
Heinong26	NE	NSP	MG I	Heilongjiang, China
Heinong33	NE	NSP	MG I	Heilongjiang, China
Heinong35	NE	NSP	MG I	Heilongjiang, China
Heinong37	NE	NSP	MG I	Heilongjiang, China
Heinong43	NE	NSP	MG I	Heilongjiang, China
Heinong44	NE	NSP	MG I	Heilongjiang, China

Variety	Region	Ecotype	MG	Source
Heinong48	NE	NSP	MG I	Heilongjiang, China
Kenfeng16	NE	NSP	MG I	Heilongjiang, China
Suinong8	NE	NSP	MG I	Heilongjiang, China
Suinong10	NE	NSP	MG I	Heilongjiang, China
Changnong4	NE	NSP	MG I	Jilin, China
Miquanhuangdou	NW	NSP	MG I	Xinjiang, China
Changjihuangdou	NW	NSP	MG I	Xinjiang, China
Zhi2	NE	NSP	MG I	Jilin, China
Zihua4	NE	NSP	MG II	Heilongjiang, China
Changnong5	NE	NSP	MG II	Jilin, China
Fengdihuang	NE	NSP	MG II	Jilin, China
Huangbaozhu	NE	NSP	MG II	Jilin, China
Jilin3	NE	NSP	MG II	Jilin, China
Jilin6	NE	NSP	MG II	Jilin, China
Jilin13	NE	NSP	MG II	Jilin, China
Jilin20	NE	NSP	MG II	Jilin, China
Jilin30	NE	NSP	MG II	Jilin, China
Jilin47	NE	NSP	MG II	Jilin, China
Jiti5	NE	NSP	MG II	Jilin, China
Jiyu57	NE	NSP	MG II	Jilin, China
Jiunong9	NE	NSP	MG II	Jilin, China
Jiunong22	NE	NSP	MG II	Jilin, China
Xiaojinhuang1	NE	NSP	MG II	Jilin, China
Zaofeng1	NE	NSP	MG II	Jilin, China
Jiti1	NE	NSP	MG II	Liaoning, China
Jin6604-24	NE	NSP	MG II	Liaoning, China
Kaiyu3	NE	NSP	MG II	Liaoning, China
Kaiyu8	NE	NSP	MG II	Liaoning, China
Kaiyu10	NE	NSP	MG II	Liaoning, China
Tiefeng3	NE	NSP	MG II	Liaoning, China
Tiefeng8	NE	NSP	MG II	Liaoning, China
Tiefeng18	NE	NSP	MG II	Liaoning, China
Tiefeng19	NE	NSP	MG II	Liaoning, China
Tiefeng20	NE	NSP	MG II	Liaoning, China
Fushou	NE	NSP	MG II	Liaoning, China
Jindou2	NE	NSP	MG II	Shanxi, China
Jindou3	NE	NSP	MG II	Shanxi, China
Dandou2	NE	NSP	MG III	Liaoning, China
Dandou4	NE	NSP	MG III	Liaoning, China
Jin8-14	NE	NSP	MG III	Liaoning, China
Jindou33	NE	NSP	MG III	Liaoning, China
Liaodou15	NE	NSP	MG III	Liaoning, China
Tiefeng31	NE	NSP	MG III	Liaoning, China
Dandou1	NE	NSP	MG IV	Liaoning, China
Tiefeng29	NE	NSP	MG IV	Liaoning, China
Changpingqingdou	NE	NSP	MG IV	Beijing, China
Huairouhuangdou	HHH	NSP	MG III	Beijing, China
Qunyingdou	HHH	NSP	MG III	Hebei, China
Zhonghuang30	HHH	NSP	MG III	Beijing, China
Zhonghuang35	HHH	NSP	MG III	Beijing, China

Variety	Region	Ecotype	MG	Source
Jindou19	HHH	NSP	MG III	Shanxi, China
Jindou21	HHH	NSP	MG III	Shanxi, China
Jindou25	HHH	NSP	MG III	Shanxi, China
Bahong1	HHH	NSP	MG IV	Hebei, China
Youbian30	HHH	NSP	MG IV	Beijing, China
Jindou23	HHH	NSP	MG IV	Shanxi, China
Hai94	HHH	NSP	MG V	Shanxi, China
Jidou7	HHH	HSU	MG II	Hebei, China
Ludou11	HHH	HSU	MG II	Shandong, China
Qihuang10	HHH	HSU	MG II	Shandong, China
Henanzaofeng1	HHH	HSU	MG II	Henan, China
Kefeng6	HHH	HSU	MG III	Beijing, China
Handou5	HHH	HSU	MG III	Hebei, China
Jidou12	HHH	HSU	MG III	Hebei, China
Jidou17	HHH	HSU	MG III	Hebei, China
Zhongdou19	HHH	HSU	MG III	Henan, China
Zhonghuang13	HHH	HSU	MG III	Beijing, China
Zhonghuang37	HHH	HSU	MG III	Beijing, China
Hedou13	HHH	HSU	MG III	Shandong, China
Heze Niumaohuang	HHH	HSU	MG III	Shandong, China
Ludou4	HHH	HSU	MG III	Shandong, China
Yanhuang1	HHH	HSU	MG III	Shandong, China
Yidu Pingdinghuang	HHH	HSU	MG III	Shandong, China
Wenfeng5	HHH	HSU	MG III	Shandong, China
Wenfeng7	HHH	HSU	MG III	Shandong, China
Shangcai Ercaopingdingshi	HHH	HSU	MG III	Henan, China
Weiqingdou	HHH	HSU	MG III	Henan, China
Zhengzhou135	HHH	HSU	MG III	Henan, China
Naiyinheidou	HHH	HSU	MG IV	Hebei, China
Haiyang Pawanqing	HHH	HSU	MG IV	Shandong, China
Yuejin4	HHH	HSU	MG IV	Shandong, China
Yudou2	HHH	HSU	MG IV	Henan, China
Yudou8	HHH	HSU	MG IV	Henan, China
Yudou22	HHH	HSU	MG IV	Henan, China
Zheng92116	HHH	HSU	MG IV	Henan, China
Xudou1	HHH	HSU	MG IV	Jiangsu, China
Xudou2	HHH	HSU	MG IV	Jiangsu, China
Xudou5	HHH	HSU	MG IV	Jiangsu, China
Xudou9	HHH	HSU	MG IV	Jiangsu, China
58-161	HHH	HSU	MG IV	Jiangsu, China
Wandou24	HHH	HSU	MG IV	Anhui, China
Yuejin5	HHH	HSU	MG V	Shandong, China
Taixingheidou	SC	SSP	MG I	Jiangsu, China
Edou8	SC	SSP	MG I	Hubei, China
Aijiaozao	SC	SSP	MG II	Hubei, China
Tianlong1	SC	SSP	MG II	Hubei, China
Guichun1	SC	SSP	MG III	Guangxi, China
Heibiqing	SC	SSP	MG III	Guangdong, China
Zigongqingpidou	SC	SSP	MG IV	Sichuan, China
Liuyuehuang	SC	SSP	MG IV	Guizhou, China

Variety	Region	Ecotype	MG	Source
Qiandou6	SC	SSP	MG IV	Guizhou, China
Jinjiang Daqingren	SC	SSP	MG IV	Fujian, China
Taiwan75 (Green 75)	SC	SSP		Taiwan, China
Guichun8	SC	SSP	MG IV	Guangxi, China
Baihuadou	SC	SSP	MG IV	Guangdong, China
Yuling Dahuangdou	SC	SSP	MG V	Guangxi, China
Juhuangdadou	SC	SSP	MG VI	Guangdong, China
Wuhua Siyuehuang	SC	SSP	MG VI	Guangdong, China
Shangyu Kanshanbai	SC	SSP	MG VIII	Zhejiang, China
Zhechun3	SC	SSP	MG VIII	Zhejiang, China
Chengliu Niumaohuang	SC	HSU	MG IV	Henan, China
Fengcheng Niupidou	SC	SSU	MG II	Jiangxi, China
Yunyizao	SC	SSU	MG IV	Hunan, China
Dian86-4	SC	SSU	MG IV	Yunnan, China
Jinning Dahuangdou	SC	SSU	MG IV	Yunnan, China
Suidaohuang	SC	SSU	MG V	Jiangsu, China
Edou2	SC	SSU	MG V	Hubei, China
Houzimao	SC	SSU	MG V	Hubei, China
Nannong493-1	SC	SSU	MG VI	Jiangsu, China
Suxie1	SC	SSU	MG VI	Jiangsu, China
Bayueqing	SC	SSU	MG VI	Hunan, China
Jingda332	SC	SSU	MG VII	Jiangsu, China
Pingguohuangdou	SC	SSU	MG VII	Guangxi, China
Nandou12	SC	SSU	MG VIII	Sichuan, China
Yangchunqingdou	SC	SAU	MG VI	Guangdong, China
Lanxi Daqingdou	SC	SAU	MG VIII	Zhejiang, China
Zigongdongdou	SC	SAU	MG VIII	Sichuan, China
Shangrao Daqingsi	SC	SAU	MG VIII	Jiangxi, China
Qiudou1	SC	SAU	MG VIII	Hunan, China
Merit	USA	USA	MG 0	USA
Clay	USA	USA	MG 0	USA
McCall	USA	USA	MG 0	USA
Norman	USA	USA	MG 0	USA
Portage	USA	USA	MG 0	USA
Wilkin	USA	USA	MG 0	USA
Chippewa64	USA	USA	MG I	USA
Evans	USA	USA	MG I	USA
Amsoy	USA	USA	MG II	USA
Amsoy71	USA	USA	MG II	USA
Beeson	USA	USA	MG II	USA
Corsoy	USA	USA	MG II	USA
Hark	USA	USA	MG II	USA
Harosoy63	USA	USA	MG II	USA
Wayne	USA	USA	MG III	USA
Clark	USA	USA	MG IV	USA
Cutler	USA	USA	MG IV	USA
Bedford	USA	USA	MG V	USA
Dare	USA	USA	MG V	USA
Forrest	USA	USA	MG V	USA
Centennial	USA	USA	MG VI	USA

Variety	Region	Ecotype	MG	Source
Hood	USA	USA	MG VI	USA
Tracy	USA	USA	MG VI	USA
Bragg	USA	USA	MG VII	USA
Braxton	USA	USA	MG VII	USA
Dowling	USA	USA	MG VII	USA
Jupiter	USA	USA	MG VIII	USA

* MG: Maturity Group；HHH: Huang-Huai-Hai; NE: Northeast China; NW: Northwest China; SC: South China. NSP: Northern Spring Planting ecotype; HSU: Huang-Huai-Hai Summer Planting ecotype; SSP: Southern Spring Planting ecotype; SSU: Southern Summer Planting ecotype; SAU: Southern Autumn Planting ecotype.

Supplementary Table S3. The haplotype combinations of *GmPRRs* among the 207 soybean varieties

Variety	*GmPRR1*	*GmPRR2*	*GmPRR3*	*GmPRR4*	*GmPRR5*	*GmPRR6*	*GmPRR7*	*GmPRR8*	*GmPRR9*	*GmPRR10*	*GmPRR11*	*GmPRR12*	*GmPRR13*	*GmPRR14*
Huajiang4	*GmPRR1*^{H1}	*GmPRR2*^{H4}	*GmPRR3*^{H1}	*GmPRR4*^{H1}	*GmPRR5*^{H1}	*GmPRR6*^{H1}	*GmPRR7*^{H2}	*GmPRR8*^{H1}	*GmPRR9*^{H1}	*GmPRR10*^{H1}	*GmPRR11*^{H1}	*GmPRR12*^{H1}	/	*GmPRR14*^{H2}
Hefeng25	*GmPRR1*^{H1}	*GmPRR2*^{H4}	*GmPRR3*^{H1}	*GmPRR4*^{H1}	*GmPRR5*^{H1}	/	*GmPRR7*^{H2}	*GmPRR8*^{H1}	*GmPRR9*^{H1}	*GmPRR10*^{H1}	*GmPRR11*^{H1}	*GmPRR12*^{H1}	*GmPRR13*^{H2}	*GmPRR14*^{H2}
Suinong14	*GmPRR1*^{H1}	*GmPRR2*^{H4}	*GmPRR3*^{H1}	*GmPRR4*^{H1}	*GmPRR5*^{H1}	*GmPRR6*^{H1}	*GmPRR7*^{H2}	*GmPRR8*^{H1}	*GmPRR9*^{H1}	*GmPRR10*^{H1}	*GmPRR11*^{H1}	*GmPRR12*^{H1}	*GmPRR13*^{H2}	*GmPRR14*^{H2}
Suinong28	*GmPRR1*^{H1}	*GmPRR2*^{H4}	*GmPRR3*^{H1}	*GmPRR4*^{H1}	*GmPRR5*^{H1}	*GmPRR6*^{H1}	*GmPRR7*^{H2}	*GmPRR8*^{H1}	*GmPRR9*^{H1}	*GmPRR10*^{H1}	*GmPRR11*^{H1}	*GmPRR12*^{H1}	*GmPRR13*^{H2}	*GmPRR14*^{H2}
Beifeng11	*GmPRR1*^{H1}	*GmPRR2*^{H4}	*GmPRR3*^{H1}	*GmPRR4*^{H1}	*GmPRR5*^{H1}	*GmPRR6*^{H1}	*GmPRR7*^{H2}	*GmPRR8*^{H1}	*GmPRR9*^{H1}	*GmPRR10*^{H1}	*GmPRR11*^{H1}	*GmPRR12*^{H3}	*GmPRR13*^{H2}	*GmPRR14*^{H2}
Beifeng9	*GmPRR1*^{H1}	*GmPRR2*^{H4}	*GmPRR3*^{H1}	*GmPRR4*^{H1}	*GmPRR5*^{H1}	*GmPRR6*^{H2}	*GmPRR7*^{H3}	*GmPRR8*^{H1}	*GmPRR9*^{H1}	*GmPRR10*^{H1}	*GmPRR11*^{H1}	*GmPRR12*^{H1}	*GmPRR13*^{H1}	*GmPRR14*^{H2}
Heihe9	*GmPRR1*^{H1}	*GmPRR2*^{H4}	*GmPRR3*^{H1}	*GmPRR4*^{H1}	*GmPRR5*^{H1}	*GmPRR6*^{H2}	*GmPRR7*^{H2}	*GmPRR8*^{H1}	*GmPRR9*^{H1}	*GmPRR10*^{H1}	*GmPRR11*^{H1}	*GmPRR12*^{H1}	*GmPRR13*^{H2}	*GmPRR14*^{H2}
Heinong35	*GmPRR1*^{H1}	*GmPRR2*^{H4}	*GmPRR3*^{H1}	*GmPRR4*^{H1}	*GmPRR5*^{H1}	*GmPRR6*^{H2}	*GmPRR7*^{H2}	*GmPRR8*^{H1}	*GmPRR9*^{H1}	*GmPRR10*^{H1}	*GmPRR11*^{H1}	*GmPRR12*^{H1}	*GmPRR13*^{H2}	/
Jilin47	*GmPRR1*^{H1}	*GmPRR2*^{H4}	*GmPRR3*^{H1}	*GmPRR4*^{H1}	*GmPRR5*^{H2}	*GmPRR6*^{H2}	*GmPRR7*^{H2}	*GmPRR8*^{H1}	*GmPRR9*^{H1}	*GmPRR10*^{H1}	*GmPRR11*^{H1}	*GmPRR12*^{H1}	*GmPRR13*^{H2}	*GmPRR14*^{H2}
Beidou5	*GmPRR1*^{H1}	*GmPRR2*^{H4}	*GmPRR3*^{H3}	/	*GmPRR5*^{H1}	*GmPRR6*^{H1}	*GmPRR7*^{H2}	*GmPRR8*^{H1}	*GmPRR9*^{H1}	*GmPRR10*^{H1}	*GmPRR11*^{H1}	*GmPRR12*^{H1}	*GmPRR13*^{H2}	*GmPRR14*^{H2}
Dwngke1	*GmPRR1*^{H1}	*GmPRR2*^{H4}	*GmPRR3*^{H3}	*GmPRR4*^{H1}	*GmPRR5*^{H1}	*GmPRR6*^{H1}	*GmPRR7*^{H2}	*GmPRR8*^{H1}	*GmPRR9*^{H1}	*GmPRR10*^{H1}	*GmPRR11*^{H1}	*GmPRR12*^{H1}	*GmPRR13*^{H2}	*GmPRR14*^{H2}
Zhonghuang35	*GmPRR1*^{H2}	*GmPRR2*^{H4}	/	*GmPRR4*^{H1}	*GmPRR5*^{H1}	*GmPRR6*^{H2}	/	/	*GmPRR9*^{H1}	*GmPRR10*^{H1}	*GmPRR11*^{H1}	/	/	*GmPRR14*^{H2}
Suinong10	*GmPRR1*^{H3}	*GmPRR2*^{H1}	*GmPRR3*^{H1}	*GmPRR4*^{H1}	*GmPRR5*^{H1}	*GmPRR6*^{H1}	*GmPRR7*^{H3}	*GmPRR8*^{H2}	*GmPRR9*^{H1}	*GmPRR10*^{H1}	*GmPRR11*^{H1}	*GmPRR12*^{H3}	*GmPRR13*^{H1}	*GmPRR14*^{H2}
Tiefeng3	*GmPRR1*^{H3}	*GmPRR2*^{H1}	*GmPRR3*^{H2}	*GmPRR4*^{H1}	*GmPRR5*^{H1}	*GmPRR6*^{H2}	*GmPRR7*^{H3}	*GmPRR8*^{H1}	*GmPRR9*^{H1}	*GmPRR10*^{H1}	*GmPRR11*^{H1}	*GmPRR12*^{H3}	*GmPRR13*^{H1}	*GmPRR14*^{H2}
Tiefeng 19	*GmPRR1*^{H3}	*GmPRR2*^{H1}	*GmPRR3*^{H2}	*GmPRR4*^{H1}	*GmPRR5*^{H1}	*GmPRR6*^{H2}	*GmPRR7*^{H3}	*GmPRR8*^{H1}	*GmPRR9*^{H1}	*GmPRR10*^{H1}	*GmPRR11*^{H1}	*GmPRR12*^{H3}	*GmPRR13*^{H1}	*GmPRR14*^{H2}
Jilin3	*GmPRR1*^{H3}	*GmPRR2*^{H1}	/	*GmPRR4*^{H1}	*GmPRR5*^{H1}	*GmPRR6*^{H2}	*GmPRR7*^{H3}	*GmPRR8*^{H1}	*GmPRR9*^{H1}	*GmPRR10*^{H1}	*GmPRR11*^{H1}	*GmPRR12*^{H3}	*GmPRR13*^{H2}	*GmPRR14*^{H2}
Jilin4	*GmPRR1*^{H3}	*GmPRR2*^{H1}	*GmPRR3*^{H2}	*GmPRR4*^{H2}	*GmPRR5*^{H1}	*GmPRR6*^{H2}	*GmPRR7*^{H3}	*GmPRR8*^{H1}	*GmPRR9*^{H1}	*GmPRR10*^{H1}	*GmPRR11*^{H1}	*GmPRR12*^{H3}	*GmPRR13*^{H2}	*GmPRR14*^{H2}
clay	*GmPRR1*^{H3}	*GmPRR2*^{H3}	*GmPRR3*^{H2}	*GmPRR4*^{H1}	*GmPRR5*^{H1}	*GmPRR6*^{H1}	*GmPRR7*^{H3}	*GmPRR8*^{H2}	*GmPRR9*^{H1}	*GmPRR10*^{H1}	*GmPRR11*^{H1}	*GmPRR12*^{H2}	*GmPRR13*^{H2}	*GmPRR14*^{H2}
PI548531	*GmPRR1*^{H3}	*GmPRR2*^{H3}	*GmPRR3*^{H2}	*GmPRR4*^{H1}	*GmPRR5*^{H1}	*GmPRR6*^{H2}	*GmPRR7*^{H3}	*GmPRR8*^{H1}	*GmPRR9*^{H1}	*GmPRR10*^{H1}	*GmPRR11*^{H1}	*GmPRR12*^{H3}	*GmPRR13*^{H2}	*GmPRR14*^{H2}
Jin8-14	*GmPRR1*^{H3}	*GmPRR2*^{H3}	*GmPRR3*^{H3}	*GmPRR4*^{H1}	*GmPRR5*^{H1}	*GmPRR6*^{H1}	*GmPRR7*^{H3}	*GmPRR8*^{H2}	*GmPRR9*^{H3}	*GmPRR10*^{H1}	*GmPRR11*^{H2}	*GmPRR12*^{H2}	*GmPRR13*^{H1}	*GmPRR14*^{H2}
Heinong44	*GmPRR1*^{H3}	*GmPRR2*^{H4}	*GmPRR3*^{H1}	*GmPRR4*^{H1}	*GmPRR5*^{H1}	*GmPRR6*^{H1}	*GmPRR7*^{H3}	*GmPRR8*^{H1}	*GmPRR9*^{H1}	*GmPRR10*^{H1}	*GmPRR11*^{H1}	*GmPRR12*^{H1}	*GmPRR13*^{H1}	*GmPRR14*^{H2}
Jidou7	*GmPRR1*^{H3}	*GmPRR2*^{H4}	*GmPRR3*^{H1}	*GmPRR4*^{H2}	*GmPRR5*^{H1}	*GmPRR6*^{H2}	*GmPRR7*^{H2}	*GmPRR8*^{H1}	*GmPRR9*^{H1}	*GmPRR10*^{H1}	/	*GmPRR12*^{H2}	*GmPRR13*^{H1}	*GmPRR14*^{H2}
Wayne	*GmPRR1*^{H3}	*GmPRR2*^{H4}	*GmPRR3*^{H1}	*GmPRR4*^{H2}	*GmPRR5*^{H1}	*GmPRR6*^{H2}	*GmPRR7*^{H3}	*GmPRR8*^{H1}	*GmPRR9*^{H1}	*GmPRR10*^{H1}	*GmPRR11*^{H1}	*GmPRR12*^{H2}	*GmPRR13*^{H2}	*GmPRR14*^{H2}

Variety	***GmPRR1***	***GmPRR2***	***GmPRR3***	***GmPRR4***	***GmPRR5***	***GmPRR6***	***GmPRR7***	***GmPRR8***	***GmPRR9***	***GmPRR10***	***GmPRR11***	***GmPRR12***	***GmPRR13***	***GmPRR14***
Qihuang10	*GmPRR1*^{H3}	*GmPRR2*^{H4}	*GmPRR3*^{H1}	*GmPRR4*^{H2}	*GmPRR5*^{H1}	*GmPRR6*^{H2}	*GmPRR7*^{H3}	*GmPRR8*^{H2}	*GmPRR9*^{H1}	*GmPRR10*^{H1}	*GmPRR11*^{H1}	*GmPRR12*^{H3}	*GmPRR13*^{H2}	*GmPRR14*^{H2}
Clark	*GmPRR1*^{H3}	*GmPRR2*^{H4}	*GmPRR3*^{H2}	*GmPRR4*^{H1}	*GmPRR5*^{H1}	*GmPRR6*^{H2}	*GmPRR7*^{H3}	*GmPRR8*^{H1}	*GmPRR9*^{H1}	*GmPRR10*^{H1}	*GmPRR11*^{H1}	*GmPRR12*^{H2}	*GmPRR13*^{H2}	*GmPRR14*^{H2}
Kaiyu3	*GmPRR1*^{H3}	*GmPRR2*^{H4}	*GmPRR3*^{H2}	*GmPRR4*^{H1}	*GmPRR5*^{H1}	*GmPRR6*^{H2}	*GmPRR7*^{H2}	*GmPRR8*^{H1}	*GmPRR9*^{H1}	*GmPRR10*^{H1}	*GmPRR11*^{H1}	*GmPRR12*^{H3}	*GmPRR13*^{H2}	*GmPRR14*^{H2}
Jindou1	*GmPRR1*^{H3}	*GmPRR2*^{H4}	*GmPRR3*^{H2}	*GmPRR4*^{H1}	*GmPRR5*^{H1}	*GmPRR6*^{H3}	*GmPRR7*^{H3}	*GmPRR8*^{H2}	*GmPRR9*^{H3}	*GmPRR10*^{H1}	*GmPRR11*^{H1}	*GmPRR12*^{H3}	*GmPRR13*^{H2}	/
Hai94	*GmPRR1*^{H3}	*GmPRR2*^{H4}	*GmPRR3*^{H2}	*GmPRR4*^{H1}	*GmPRR5*^{H1}	*GmPRR6*^{H3}	*GmPRR7*^{H3}	*GmPRR8*^{H2}	*GmPRR9*^{H3}	*GmPRR10*^{H1}	*GmPRR11*^{H1}	*GmPRR12*^{H3}	*GmPRR13*^{H2}	*GmPRR14*^{H3}
Jidou12	*GmPRR1*^{H3}	*GmPRR2*^{H4}	*GmPRR3*^{H2}	*GmPRR4*^{H2}	*GmPRR5*^{H2}	*GmPRR6*^{H4}	*GmPRR7*^{H3}	*GmPRR8*^{H1}	*GmPRR9*^{H3}	*GmPRR10*^{H1}	*GmPRR11*^{H1}	*GmPRR12*^{H4}	*GmPRR13*^{H2}	*GmPRR14*^{H2}
Suxie1	*GmPRR1*^{H3}	*GmPRR2*^{H4}	*GmPRR3*^{H3}	*GmPRR4*^{H1}	/	*GmPRR6*^{H1}	*GmPRR7*^{H3}	*GmPRR8*^{H1}	*GmPRR9*^{H4}	*GmPRR10*^{H1}	*GmPRR11*^{H2}	*GmPRR12*^{H4}	*GmPRR13*^{H2}	*GmPRR14*^{H2}
Jinyuan2	*GmPRR1*^{H3}	*GmPRR2*^{H4}	*GmPRR3*^{H3}	*GmPRR4*^{H1}	*GmPRR5*^{H1}	*GmPRR6*^{H1}	*GmPRR7*^{H3}	*GmPRR8*^{H2}	*GmPRR9*^{H1}	*GmPRR10*^{H1}	*GmPRR11*^{H1}	*GmPRR12*^{H1}	*GmPRR13*^{H2}	*GmPRR14*^{H2}
Jilin20	*GmPRR1*^{H3}	*GmPRR2*^{H4}	*GmPRR3*^{H3}	*GmPRR4*^{H1}	*GmPRR5*^{H1}	*GmPRR6*^{H2}	*GmPRR7*^{H2}	*GmPRR8*^{H1}	*GmPRR9*^{H1}	*GmPRR10*^{H1}	*GmPRR11*^{H1}	*GmPRR12*^{H3}	*GmPRR13*^{H1}	*GmPRR14*^{H2}
Changnong5	*GmPRR1*^{H3}	*GmPRR2*^{H4}	*GmPRR3*^{H3}	*GmPRR4*^{H1}	*GmPRR5*^{H1}	*GmPRR6*^{H2}	*GmPRR7*^{H2}	*GmPRR8*^{H1}	*GmPRR9*^{H1}	*GmPRR10*^{H1}	*GmPRR11*^{H1}	*GmPRR12*^{H3}	*GmPRR13*^{H1}	*GmPRR14*^{H2}
Changnong4	*GmPRR1*^{H3}	*GmPRR2*^{H4}	*GmPRR3*^{H3}	*GmPRR4*^{H1}	*GmPRR5*^{H1}	*GmPRR6*^{H2}	*GmPRR7*^{H3}	*GmPRR8*^{H1}	*GmPRR9*^{H1}	*GmPRR10*^{H1}	*GmPRR11*^{H1}	*GmPRR12*^{H3}	*GmPRR13*^{H1}	*GmPRR14*^{H2}
Jilin30	*GmPRR1*^{H3}	*GmPRR2*^{H4}	*GmPRR3*^{H3}	*GmPRR4*^{H1}	*GmPRR5*^{H1}	*GmPRR6*^{H2}	*GmPRR7*^{H3}	*GmPRR8*^{H1}	*GmPRR9*^{H1}	*GmPRR10*^{H1}	*GmPRR11*^{H1}	*GmPRR12*^{H3}	*GmPRR13*^{H1}	*GmPRR14*^{H2}
Zheng92116	*GmPRR1*^{H3}	*GmPRR2*^{H4}	*GmPRR3*^{H3}	*GmPRR4*^{H2}	*GmPRR5*^{H1}	*GmPRR6*^{H1}	*GmPRR7*^{H3}	/	*GmPRR9*^{H1}	*GmPRR10*^{H1}	*GmPRR11*^{H2}	*GmPRR12*^{H3}	*GmPRR13*^{H1}	*GmPRR14*^{H2}
Edou8	*GmPRR1*^{H3}	*GmPRR2*^{H4}	*GmPRR3*^{H3}	*GmPRR4*^{H2}	*GmPRR5*^{H1}	*GmPRR6*^{H1}	*GmPRR7*^{H3}	*GmPRR8*^{H1}	*GmPRR9*^{H3}	*GmPRR10*^{H1}	*GmPRR11*^{H2}	*GmPRR12*^{H1}	*GmPRR13*^{H2}	*GmPRR14*^{H2}
Handou5	*GmPRR1*^{H3}	*GmPRR2*^{H4}	*GmPRR3*^{H3}	*GmPRR4*^{H2}	*GmPRR5*^{H1}	*GmPRR6*^{H2}	*GmPRR7*^{H3}	*GmPRR8*^{H1}	*GmPRR9*^{H1}	*GmPRR10*^{H1}	*GmPRR11*^{H2}	*GmPRR12*^{H2}	*GmPRR13*^{H2}	*GmPRR14*^{H2}
Haiyangpamanqing	*GmPRR1*^{H3}	*GmPRR2*^{H4}	*GmPRR3*^{H3}	*GmPRR4*^{H2}	*GmPRR5*^{H2}	*GmPRR6*^{H2}	*GmPRR7*^{H3}	*GmPRR8*^{H2}	*GmPRR9*^{H4}	*GmPRR10*^{H1}	*GmPRR11*^{H2}	*GmPRR12*^{H3}	*GmPRR13*^{H3}	*GmPRR14*^{H2}
Zhengzhou135	*GmPRR1*^{H3}	*GmPRR2*^{H4}	*GmPRR3*^{H3}	*GmPRR4*^{H2}	*GmPRR5*^{H4}	*GmPRR6*^{H2}	*GmPRR7*^{H3}	*GmPRR8*^{H2}	*GmPRR9*^{H1}	*GmPRR10*^{H1}	*GmPRR11*^{H1}	*GmPRR12*^{H1}	*GmPRR13*^{H2}	*GmPRR14*^{H2}
Aijiaozao	*GmPRR1*^{H3}	*GmPRR2*^{H4}	*GmPRR3*^{H3}	*GmPRR4*^{H2}	*GmPRR5*^{H4}	*GmPRR6*^{H3}	*GmPRR7*^{H2}	*GmPRR8*^{H1}	*GmPRR9*^{H1}	*GmPRR10*^{H1}	*GmPRR11*^{H2}	/	*GmPRR13*^{H2}	*GmPRR14*^{H2}
Zhonghuang37	*GmPRR1*^{H3}	*GmPRR2*^{H4}	*GmPRR3*^{H3}	*GmPRR4*^{H2}	*GmPRR5*^{H4}	*GmPRR6*^{H4}	*GmPRR7*^{H3}	*GmPRR8*^{H2}	*GmPRR9*^{H1}	*GmPRR10*^{H1}	*GmPRR11*^{H1}	*GmPRR12*^{H1}	*GmPRR13*^{H2}	*GmPRR14*^{H2}
Shangyukanshangbai	*GmPRR1*^{H3}	*GmPRR2*^{H4}	*GmPRR3*^{H3}	*GmPRR4*^{H2}	*GmPRR5*^{H4}	*GmPRR6*^{H4}	*GmPRR7*^{H4}	*GmPRR8*^{H2}	*GmPRR9*^{H2}	*GmPRR10*^{H1}	*GmPRR11*^{H2}	*GmPRR12*^{H3}	*GmPRR13*^{H2}	*GmPRR14*^{H3}
Yudou22	*GmPRR1*^{H3}	*GmPRR2*^{H4}	*GmPRR3*^{H3}	*GmPRR4*^{H2}	/	*GmPRR6*^{H1}	*GmPRR7*^{H3}	*GmPRR8*^{H2}	*GmPRR9*^{H1}	*GmPRR10*^{H1}	*GmPRR11*^{H2}	*GmPRR12*^{H4}	*GmPRR13*^{H1}	*GmPRR14*^{H2}
Wandou24	*GmPRR1*^{H3}	*GmPRR2*^{H4}	/	*GmPRR4*^{H2}	/	/	*GmPRR7*^{H3}	*GmPRR8*^{H2}	*GmPRR9*^{H1}	*GmPRR10*^{H1}	/	*GmPRR12*^{H3}	*GmPRR13*^{H2}	*GmPRR14*^{H2}
Nannong493-1	*GmPRR1*^{H3}	*GmPRR2*^{H5}	*GmPRR3*^{H3}	*GmPRR4*^{H1}	*GmPRR5*^{H2}	*GmPRR6*^{H1}	*GmPRR7*^{H3}	*GmPRR8*^{H1}	*GmPRR9*^{H2}	*GmPRR10*^{H1}	*GmPRR11*^{H2}	*GmPRR12*^{H1}	*GmPRR13*^{H2}	*GmPRR14*^{H2}
Zhongdou19	*GmPRR1*^{H3}	*GmPRR2*^{H5}	*GmPRR3*^{H3}	*GmPRR4*^{H2}	*GmPRR5*^{H2}	*GmPRR6*^{H2}	*GmPRR7*^{H2}	*GmPRR8*^{H3}	*GmPRR9*^{H1}	*GmPRR10*^{H1}	*GmPRR11*^{H2}	*GmPRR12*^{H4}	*GmPRR13*^{H2}	*GmPRR14*^{H2}

**Variety**	***GmPRR1***	***GmPRR2***	***GmPRR3***	***GmPRR4***	***GmPRR5***	***GmPRR6***	***GmPRR7***	***GmPRR8***	***GmPRR9***	***GmPRR10***	***GmPRR11***	***GmPRR12***	***GmPRR13***	***GmPRR14***
Zhechun3	*GmPRR1*^{H3}	*GmPRR2*^{H5}	*GmPRR3*^{H4}	*GmPRR4*^{H2}	*GmPRR5*^{H4}	*GmPRR6*^{H4}	*GmPRR7*^{H3}	*GmPRR8*^{H1}	*GmPRR9*^{H4}	*GmPRR10*^{H1}	*GmPRR11*^{H2}	*GmPRR12*^{H1}	*GmPRR13*^{H1}	*GmPRR14*^{H2}
Yidupingdinghuang	*GmPRR1*^{H3}	*GmPRR2*^{H6}	*GmPRR3*^{H2}	*GmPRR4*^{H3}	*GmPRR5*^{H1}	*GmPRR6*^{H2}	/	*GmPRR8*^{H1}	/	*GmPRR10*^{H1}	*GmPRR11*^{H2}	*GmPRR12*^{H1}	*GmPRR13*^{H2}	*GmPRR14*^{H2}
Fengshouhuang1	*GmPRR1*^{H3}	*GmPRR2*^{H6}	*GmPRR3*^{H2}	*GmPRR4*^{H2}	*GmPRR5*^{H1}	*GmPRR6*^{H2}	/	*GmPRR8*^{H3}	*GmPRR9*^{H3}	*GmPRR10*^{H1}	*GmPRR11*^{H2}	*GmPRR12*^{H1}	*GmPRR13*^{H3}	*GmPRR14*^{H2}
Jindou21	*GmPRR1*^{H3}	*GmPRR2*^{H6}	*GmPRR3*^{H2}	*GmPRR4*^{H1}	*GmPRR5*^{H1}	*GmPRR6*^{H2}	*GmPRR7*^{H3}	*GmPRR8*^{H2}	/	*GmPRR10*^{H1}	*GmPRR11*^{H1}	*GmPRR12*^{H1}	*GmPRR13*^{H2}	*GmPRR14*^{H3}
Henanzaofeng1	*GmPRR1*^{H3}	*GmPRR2*^{H6}	*GmPRR3*^{H3}	*GmPRR4*^{H2}	*GmPRR5*^{H1}	*GmPRR6*^{H2}	*GmPRR7*^{H3}	*GmPRR8*^{H1}	/	*GmPRR10*^{H1}	*GmPRR11*^{H2}	*GmPRR12*^{H2}	/	*GmPRR14*^{H2}
Mengdou30	*GmPRR1*^{H3}	/	/	/	*GmPRR5*^{H1}	*GmPRR6*^{H2}	/	/	*GmPRR9*^{H1}	*GmPRR10*^{H1}	*GmPRR11*^{H1}	*GmPRR12*^{H3}	*GmPRR13*^{H1}	*GmPRR14*^{H2}
Suinong3	*GmPRR1*^{H4}	*GmPRR2*^{H1}	*GmPRR3*^{H1}	*GmPRR4*^{H1}	*GmPRR5*^{H1}	*GmPRR6*^{H1}	*GmPRR7*^{H3}	*GmPRR8*^{H1}	*GmPRR9*^{H1}	*GmPRR10*^{H1}	*GmPRR11*^{H1}	*GmPRR12*^{H1}	*GmPRR13*^{H2}	*GmPRR14*^{H2}
Kexi283	*GmPRR1*^{H4}	*GmPRR2*^{H1}	*GmPRR3*^{H1}	*GmPRR4*^{H1}	*GmPRR5*^{H1}	*GmPRR6*^{H1}	*GmPRR7*^{H3}	*GmPRR8*^{H2}	*GmPRR9*^{H1}	*GmPRR10*^{H1}	*GmPRR11*^{H1}	*GmPRR12*^{H1}	*GmPRR13*^{H2}	*GmPRR14*^{H2}
Kenfeng16	*GmPRR1*^{H4}	*GmPRR2*^{H1}	*GmPRR3*^{H1}	*GmPRR4*^{H1}	*GmPRR5*^{H1}	*GmPRR6*^{H2}	/	*GmPRR8*^{H1}	*GmPRR9*^{H1}	*GmPRR10*^{H1}	*GmPRR11*^{H1}	*GmPRR12*^{H3}	*GmPRR13*^{H2}	*GmPRR14*^{H2}
Mancangjin	*GmPRR1*^{H4}	*GmPRR2*^{H1}	*GmPRR3*^{H2}	*GmPRR4*^{H1}	*GmPRR5*^{H1}	*GmPRR6*^{H1}	*GmPRR7*^{H3}	*GmPRR8*^{H1}	*GmPRR9*^{H1}	*GmPRR10*^{H1}	*GmPRR11*^{H1}	*GmPRR12*^{H3}	*GmPRR13*^{H1}	*GmPRR14*^{H2}
Jingshanpu	*GmPRR1*^{H4}	*GmPRR2*^{H1}	*GmPRR3*^{H2}	*GmPRR4*^{H1}	*GmPRR5*^{H1}	*GmPRR6*^{H1}	*GmPRR7*^{H3}	*GmPRR8*^{H1}	*GmPRR9*^{H1}	*GmPRR10*^{H1}	*GmPRR11*^{H1}	*GmPRR12*^{H3}	*GmPRR13*^{H1}	*GmPRR14*^{H2}
Huangbaozhu	*GmPRR1*^{H4}	*GmPRR2*^{H1}	*GmPRR3*^{H2}	*GmPRR4*^{H1}	*GmPRR5*^{H1}	*GmPRR6*^{H2}	*GmPRR7*^{H3}	*GmPRR8*^{H1}	*GmPRR9*^{H1}	*GmPRR10*^{H1}	*GmPRR11*^{H1}	*GmPRR12*^{H3}	*GmPRR13*^{H1}	*GmPRR14*^{H2}
Tiefeng18	*GmPRR1*^{H4}	*GmPRR2*^{H1}	*GmPRR3*^{H3}	*GmPRR4*^{H1}	*GmPRR5*^{H1}	*GmPRR6*^{H1}	/	*GmPRR8*^{H1}	*GmPRR9*^{H1}	*GmPRR10*^{H1}	*GmPRR11*^{H2}	*GmPRR12*^{H3}	*GmPRR13*^{H1}	/
Xiaojinhuang1	*GmPRR1*^{H4}	*GmPRR2*^{H1}	*GmPRR3*^{H3}	*GmPRR4*^{H1}	*GmPRR5*^{H1}	*GmPRR6*^{H2}	*GmPRR7*^{H3}	*GmPRR8*^{H1}	*GmPRR9*^{H1}	*GmPRR10*^{H1}	*GmPRR11*^{H1}	*GmPRR12*^{H3}	*GmPRR13*^{H1}	*GmPRR14*^{H2}
Jiti1	*GmPRR1*^{H4}	*GmPRR2*^{H1}	*GmPRR3*^{H3}	*GmPRR4*^{H1}	*GmPRR5*^{H1}	*GmPRR6*^{H2}	*GmPRR7*^{H3}	*GmPRR8*^{H1}	*GmPRR9*^{H1}	*GmPRR10*^{H1}	*GmPRR11*^{H1}	*GmPRR12*^{H3}	*GmPRR13*^{H1}	*GmPRR14*^{H2}
Jilin6	*GmPRR1*^{H4}	*GmPRR2*^{H1}	*GmPRR3*^{H3}	*GmPRR4*^{H1}	*GmPRR5*^{H1}	*GmPRR6*^{H2}	*GmPRR7*^{H3}	*GmPRR8*^{H1}	*GmPRR9*^{H1}	*GmPRR10*^{H1}	*GmPRR11*^{H1}	*GmPRR12*^{H3}	*GmPRR13*^{H1}	*GmPRR14*^{H2}
Jin6604-24	*GmPRR1*^{H4}	*GmPRR2*^{H1}	*GmPRR3*^{H3}	*GmPRR4*^{H1}	*GmPRR5*^{H1}	*GmPRR6*^{H2}	*GmPRR7*^{H3}	*GmPRR8*^{H1}	*GmPRR9*^{H1}	*GmPRR10*^{H1}	*GmPRR11*^{H1}	*GmPRR12*^{H3}	*GmPRR13*^{H1}	*GmPRR14*^{H2}
Jin33	*GmPRR1*^{H4}	*GmPRR2*^{H1}	*GmPRR3*^{H3}	*GmPRR4*^{H1}	*GmPRR5*^{H1}	*GmPRR6*^{H2}	*GmPRR7*^{H3}	*GmPRR8*^{H2}	*GmPRR9*^{H1}	*GmPRR10*^{H1}	*GmPRR11*^{H1}	*GmPRR12*^{H3}	*GmPRR13*^{H1}	*GmPRR14*^{H2}
Dandou4	*GmPRR1*^{H4}	*GmPRR2*^{H3}	*GmPRR3*^{H1}	*GmPRR4*^{H1}	*GmPRR5*^{H1}	*GmPRR6*^{H2}	*GmPRR7*^{H2}	*GmPRR8*^{H2}	*GmPRR9*^{H1}	*GmPRR10*^{H1}	*GmPRR11*^{H1}	*GmPRR12*^{H1}	*GmPRR13*^{H1}	*GmPRR14*^{H2}
Hefeng35	*GmPRR1*^{H4}	*GmPRR2*^{H4}	*GmPRR3*^{H1}	*GmPRR4*^{H1}	*GmPRR5*^{H1}	*GmPRR6*^{H1}	*GmPRR7*^{H2}	*GmPRR8*^{H1}	*GmPRR9*^{H1}	*GmPRR10*^{H1}	*GmPRR11*^{H1}	*GmPRR12*^{H1}	*GmPRR13*^{H1}	*GmPRR14*^{H2}
Hefeng47	*GmPRR1*^{H4}	*GmPRR2*^{H4}	*GmPRR3*^{H1}	*GmPRR4*^{H1}	*GmPRR5*^{H1}	*GmPRR6*^{H1}	*GmPRR7*^{H2}	*GmPRR8*^{H1}	*GmPRR9*^{H1}	*GmPRR10*^{H1}	*GmPRR11*^{H1}	*GmPRR12*^{H1}	*GmPRR13*^{H1}	*GmPRR14*^{H2}
Hefeng50	*GmPRR1*^{H4}	*GmPRR2*^{H4}	*GmPRR3*^{H1}	*GmPRR4*^{H1}	*GmPRR5*^{H1}	*GmPRR6*^{H1}	*GmPRR7*^{H2}	*GmPRR8*^{H1}	*GmPRR9*^{H1}	*GmPRR10*^{H1}	*GmPRR11*^{H1}	*GmPRR12*^{H1}	*GmPRR13*^{H1}	*GmPRR14*^{H2}
Fushou	*GmPRR1*^{H4}	*GmPRR2*^{H4}	*GmPRR3*^{H1}	*GmPRR4*^{H1}	*GmPRR5*^{H1}	*GmPRR6*^{H1}	*GmPRR7*^{H2}	*GmPRR8*^{H1}	*GmPRR9*^{H1}	*GmPRR10*^{H1}	*GmPRR11*^{H1}	*GmPRR12*^{H1}	*GmPRR13*^{H1}	*GmPRR14*^{H2}
Heihe27	*GmPRR1*^{H4}	*GmPRR2*^{H4}	*GmPRR3*^{H1}	*GmPRR4*^{H1}	*GmPRR5*^{H1}	*GmPRR6*^{H1}	*GmPRR7*^{H3}	*GmPRR8*^{H1}	*GmPRR9*^{H1}	*GmPRR10*^{H1}	*GmPRR11*^{H1}	/	*GmPRR13*^{H2}	*GmPRR14*^{H2}

**Variety**	***GmPRR1***	***GmPRR2***	***GmPRR3***	***GmPRR4***	***GmPRR5***	***GmPRR6***	***GmPRR7***	***GmPRR8***	***GmPRR9***	***GmPRR10***	***GmPRR11***	***GmPRR12***	***GmPRR13***	***GmPRR14***
Dongnong4	*GmPRR1*^{H4}	*GmPRR2*^{H4}	*GmPRR3*^{H1}	*GmPRR4*^{H1}	*GmPRR5*^{H1}	*GmPRR6*^{H1}	*GmPRR7*^{H3}	*GmPRR8*^{H1}	*GmPRR9*^{H1}	*GmPRR10*^{H1}	*GmPRR11*^{H1}	*GmPRR12*^{H1}	*GmPRR13*^{H1}	*GmPRR14*^{H2}
Hejiao8	*GmPRR1*^{H4}	*GmPRR2*^{H4}	*GmPRR3*^{H1}	*GmPRR4*^{H1}	*GmPRR5*^{H1}	*GmPRR6*^{H1}	*GmPRR7*^{H3}	*GmPRR8*^{H1}	*GmPRR9*^{H1}	*GmPRR10*^{H1}	*GmPRR11*^{H1}	*GmPRR12*^{H1}	*GmPRR13*^{H1}	*GmPRR14*^{H2}
Suinong8	*GmPRR1*^{H4}	*GmPRR2*^{H4}	*GmPRR3*^{H1}	*GmPRR4*^{H1}	*GmPRR5*^{H1}	*GmPRR6*^{H1}	*GmPRR7*^{H3}	*GmPRR8*^{H1}	*GmPRR9*^{H1}	*GmPRR10*^{H1}	*GmPRR11*^{H1}	*GmPRR12*^{H1}	*GmPRR13*^{H1}	*GmPRR14*^{H2}
Heinong43	*GmPRR1*^{H4}	*GmPRR2*^{H4}	*GmPRR3*^{H1}	*GmPRR4*^{H1}	*GmPRR5*^{H1}	*GmPRR6*^{H1}	*GmPRR7*^{H3}	*GmPRR8*^{H1}	*GmPRR9*^{H1}	*GmPRR10*^{H1}	*GmPRR11*^{H1}	*GmPRR12*^{H1}	*GmPRR13*^{H1}	*GmPRR14*^{H2}
Beifneg2	*GmPRR1*^{H4}	*GmPRR2*^{H4}	*GmPRR3*^{H1}	*GmPRR4*^{H1}	*GmPRR5*^{H1}	*GmPRR6*^{H1}	*GmPRR7*^{H3}	*GmPRR8*^{H1}	*GmPRR9*^{H1}	*GmPRR10*^{H1}	*GmPRR11*^{H1}	*GmPRR12*^{H1}	*GmPRR13*^{H2}	*GmPRR14*^{H2}
Beihudou	*GmPRR1*^{H4}	*GmPRR2*^{H4}	*GmPRR3*^{H1}	*GmPRR4*^{H1}	*GmPRR5*^{H1}	*GmPRR6*^{H1}	*GmPRR7*^{H3}	*GmPRR8*^{H1}	*GmPRR9*^{H1}	*GmPRR10*^{H1}	*GmPRR11*^{H1}	*GmPRR12*^{H1}	*GmPRR13*^{H2}	*GmPRR14*^{H2}
PI 548540	*GmPRR1*^{H4}	*GmPRR2*^{H4}	*GmPRR3*^{H1}	*GmPRR4*^{H1}	*GmPRR5*^{H1}	*GmPRR6*^{H1}	*GmPRR7*^{H3}	*GmPRR8*^{H1}	*GmPRR9*^{H1}	*GmPRR10*^{H1}	*GmPRR11*^{H1}	*GmPRR12*^{H3}	*GmPRR13*^{H2}	*GmPRR14*^{H2}
Harosoy63	*GmPRR1*^{H4}	*GmPRR2*^{H4}	*GmPRR3*^{H1}	*GmPRR4*^{H1}	*GmPRR5*^{H1}	*GmPRR6*^{H1}	*GmPRR7*^{H3}	*GmPRR8*^{H1}	*GmPRR9*^{H1}	*GmPRR10*^{H1}	*GmPRR11*^{H1}	*GmPRR12*^{H3}	*GmPRR13*^{H2}	*GmPRR14*^{H2}
Hefeng55	*GmPRR1*^{H4}	*GmPRR2*^{H4}	*GmPRR3*^{H1}	*GmPRR4*^{H1}	*GmPRR5*^{H1}	*GmPRR6*^{H1}	*GmPRR7*^{H3}	*GmPRR8*^{H1}	*GmPRR9*^{H1}	*GmPRR10*^{H1}	*GmPRR11*^{H1}	*GmPRR12*^{H3}	*GmPRR13*^{H2}	*GmPRR14*^{H2}
Changjihuangdou	*GmPRR1*^{H4}	*GmPRR2*^{H4}	*GmPRR3*^{H1}	*GmPRR4*^{H1}	*GmPRR5*^{H1}	*GmPRR6*^{H1}	*GmPRR7*^{H3}	*GmPRR8*^{H1}	*GmPRR9*^{H1}	*GmPRR10*^{H1}	*GmPRR11*^{H1}	*GmPRR12*^{H3}	*GmPRR13*^{H1}	*GmPRR14*^{H2}
Amsoy	*GmPRR1*^{H4}	*GmPRR2*^{H4}	*GmPRR3*^{H1}	*GmPRR4*^{H1}	*GmPRR5*^{H1}	*GmPRR6*^{H1}	*GmPRR7*^{H3}	*GmPRR8*^{H2}	*GmPRR9*^{H1}	*GmPRR10*^{H1}	*GmPRR11*^{H1}	*GmPRR12*^{H3}	*GmPRR13*^{H1}	*GmPRR14*^{H2}
Amsoy71	*GmPRR1*^{H4}	*GmPRR2*^{H4}	*GmPRR3*^{H1}	*GmPRR4*^{H1}	*GmPRR5*^{H1}	*GmPRR6*^{H1}	*GmPRR7*^{H3}	*GmPRR8*^{H2}	*GmPRR9*^{H1}	*GmPRR10*^{H1}	*GmPRR11*^{H1}	*GmPRR12*^{H3}	*GmPRR13*^{H1}	*GmPRR14*^{H2}
Fengshou19	*GmPRR1*^{H4}	*GmPRR2*^{H4}	*GmPRR3*^{H1}	*GmPRR4*^{H1}	*GmPRR5*^{H1}	*GmPRR6*^{H2}	*GmPRR7*^{H3}	*GmPRR8*^{H1}	*GmPRR9*^{H1}	*GmPRR10*^{H1}	*GmPRR11*^{H1}	*GmPRR12*^{H1}	*GmPRR13*^{H1}	*GmPRR14*^{H2}
Heihe38	*GmPRR1*^{H4}	*GmPRR2*^{H4}	*GmPRR3*^{H1}	*GmPRR4*^{H1}	*GmPRR5*^{H1}	*GmPRR6*^{H2}	*GmPRR7*^{H2}	*GmPRR8*^{H1}	*GmPRR9*^{H1}	*GmPRR10*^{H1}	*GmPRR11*^{H1}	*GmPRR12*^{H1}	*GmPRR13*^{H2}	*GmPRR14*^{H2}
Wilkin	*GmPRR1*^{H4}	*GmPRR2*^{H4}	*GmPRR3*^{H1}	*GmPRR4*^{H2}	*GmPRR5*^{H1}	*GmPRR6*^{H1}	*GmPRR7*^{H3}	*GmPRR8*^{H1}	*GmPRR9*^{H1}	*GmPRR10*^{H1}	*GmPRR11*^{H1}	*GmPRR12*^{H3}	*GmPRR13*^{H1}	*GmPRR14*^{H3}
Heihe3	*GmPRR1*^{H4}	*GmPRR2*^{H4}	*GmPRR3*^{H1}	*GmPRR4*^{H2}	*GmPRR5*^{H1}	*GmPRR6*^{H1}	*GmPRR7*^{H3}	*GmPRR8*^{H2}	*GmPRR9*^{H1}	*GmPRR10*^{H1}	*GmPRR11*^{H1}	*GmPRR12*^{H3}	*GmPRR13*^{H2}	*GmPRR14*^{H1}
Heihe51	*GmPRR1*^{H4}	*GmPRR2*^{H4}	*GmPRR3*^{H1}	*GmPRR4*^{H2}	*GmPRR5*^{H1}	*GmPRR6*^{H1}	*GmPRR7*^{H3}	*GmPRR8*^{H2}	*GmPRR9*^{H1}	*GmPRR10*^{H1}	*GmPRR11*^{H1}	*GmPRR12*^{H3}	*GmPRR13*^{H2}	*GmPRR14*^{H1}
Heihe54	*GmPRR1*^{H4}	*GmPRR2*^{H4}	*GmPRR3*^{H1}	*GmPRR4*^{H2}	*GmPRR5*^{H1}	*GmPRR6*^{H1}	*GmPRR7*^{H3}	*GmPRR8*^{H2}	*GmPRR9*^{H1}	*GmPRR10*^{H1}	*GmPRR11*^{H1}	*GmPRR12*^{H3}	*GmPRR13*^{H2}	*GmPRR14*^{H1}
Heilongjiang41	*GmPRR1*^{H4}	*GmPRR2*^{H4}	*GmPRR3*^{H1}	*GmPRR4*^{H2}	*GmPRR5*^{H1}	*GmPRR6*^{H2}	*GmPRR7*^{H3}	*GmPRR8*^{H2}	*GmPRR9*^{H1}	*GmPRR10*^{H1}	*GmPRR11*^{H1}	*GmPRR12*^{H1}	*GmPRR13*^{H2}	*GmPRR14*^{H2}
Fengshou24	*GmPRR1*^{H4}	*GmPRR2*^{H4}	*GmPRR3*^{H2}	*GmPRR4*^{H1}	*GmPRR5*^{H1}	*GmPRR6*^{H1}	*GmPRR7*^{H3}	/	*GmPRR9*^{H1}	*GmPRR10*^{H1}	*GmPRR11*^{H1}	*GmPRR12*^{H1}	*GmPRR13*^{H1}	*GmPRR14*^{H2}
Heinong16	*GmPRR1*^{H4}	*GmPRR2*^{H4}	*GmPRR3*^{H2}	*GmPRR4*^{H1}	*GmPRR5*^{H1}	*GmPRR6*^{H1}	*GmPRR7*^{H3}	*GmPRR8*^{H1}	*GmPRR9*^{H1}	*GmPRR10*^{H1}	*GmPRR11*^{H1}	*GmPRR12*^{H1}	*GmPRR13*^{H1}	*GmPRR14*^{H2}
Heinong26	*GmPRR1*^{H4}	*GmPRR2*^{H4}	*GmPRR3*^{H2}	*GmPRR4*^{H1}	*GmPRR5*^{H1}	*GmPRR6*^{H1}	*GmPRR7*^{H3}	*GmPRR8*^{H1}	*GmPRR9*^{H1}	*GmPRR10*^{H1}	*GmPRR11*^{H1}	*GmPRR12*^{H1}	*GmPRR13*^{H1}	*GmPRR14*^{H2}
Hefeng22	*GmPRR1*^{H4}	*GmPRR2*^{H4}	*GmPRR3*^{H2}	*GmPRR4*^{H1}	*GmPRR5*^{H1}	*GmPRR6*^{H1}	*GmPRR7*^{H3}	*GmPRR8*^{H1}	*GmPRR9*^{H1}	*GmPRR10*^{H1}	*GmPRR11*^{H1}	/	/	*GmPRR14*^{H2}
Heinong33	*GmPRR1*^{H4}	*GmPRR2*^{H4}	*GmPRR3*^{H2}	*GmPRR4*^{H1}	*GmPRR5*^{H1}	*GmPRR6*^{H1}	*GmPRR7*^{H3}	*GmPRR8*^{H1}	*GmPRR9*^{H1}	*GmPRR10*^{H1}	*GmPRR11*^{H1}	*GmPRR12*^{H2}	*GmPRR13*^{H2}	*GmPRR14*^{H2}
Zhi2	*GmPRR1*^{H4}	*GmPRR2*^{H4}	*GmPRR3*^{H2}	*GmPRR4*^{H1}	*GmPRR5*^{H1}	*GmPRR6*^{H1}	*GmPRR7*^{H3}	*GmPRR8*^{H1}	*GmPRR9*^{H1}	*GmPRR10*^{H1}	*GmPRR11*^{H1}	*GmPRR12*^{H3}	*GmPRR13*^{H1}	*GmPRR14*^{H2}

Variety	***GmPRR1***	***GmPRR2***	***GmPRR3***	***GmPRR4***	***GmPRR5***	***GmPRR6***	***GmPRR7***	***GmPRR8***	***GmPRR9***	***GmPRR10***	***GmPRR11***	***GmPRR12***	***GmPRR13***	***GmPRR14***
Hejiao6	$GmPRR1^{H4}$	$GmPRR2^{H4}$	$GmPRR3^{H2}$	$GmPRR4^{H1}$	$GmPRR5^{H1}$	$GmPRR6^{H1}$	$GmPRR7^{H3}$	$GmPRR8^{H1}$	$GmPRR9^{H1}$	$GmPRR10^{H1}$	$GmPRR11^{H1}$	$GmPRR12^{H3}$	$GmPRR13^{H2}$	$GmPRR14^{H2}$
McCall	$GmPRR1^{H4}$	$GmPRR2^{H4}$	$GmPRR3^{H2}$	$GmPRR4^{H1}$	$GmPRR5^{H1}$	$GmPRR6^{H1}$	$GmPRR7^{H3}$	$GmPRR8^{H1}$	$GmPRR9^{H1}$	$GmPRR10^{H1}$	$GmPRR11^{H1}$	$GmPRR12^{H3}$	$GmPRR13^{H2}$	$GmPRR14^{H2}$
Hark	$GmPRR1^{H4}$	$GmPRR2^{H4}$	$GmPRR3^{H2}$	$GmPRR4^{H1}$	$GmPRR5^{H1}$	$GmPRR6^{H1}$	$GmPRR7^{H3}$	$GmPRR8^{H1}$	$GmPRR9^{H1}$	$GmPRR10^{H1}$	$GmPRR11^{H1}$	$GmPRR12^{H3}$	$GmPRR13^{H2}$	$GmPRR14^{H2}$
Jindou2	$GmPRR1^{H4}$	$GmPRR2^{H4}$	$GmPRR3^{H2}$	$GmPRR4^{H1}$	$GmPRR5^{H1}$	$GmPRR6^{H2}$	$GmPRR7^{H3}$	$GmPRR8^{H1}$	$GmPRR9^{H1}$	$GmPRR10^{H1}$	$GmPRR11^{H1}$	$GmPRR12^{H1}$	$GmPRR13^{H2}$	$GmPRR14^{H2}$
Jiunong9	$GmPRR1^{H4}$	$GmPRR2^{H4}$	$GmPRR3^{H2}$	$GmPRR4^{H1}$	$GmPRR5^{H1}$	$GmPRR6^{H2}$	$GmPRR7^{H3}$	$GmPRR8^{H1}$	$GmPRR9^{H1}$	$GmPRR10^{H1}$	$GmPRR11^{H1}$	$GmPRR12^{H3}$	$GmPRR13^{H1}$	$GmPRR14^{H2}$
Tiefeng20	$GmPRR1^{H4}$	$GmPRR2^{H4}$	$GmPRR3^{H2}$	$GmPRR4^{H1}$	$GmPRR5^{H1}$	$GmPRR6^{H2}$	$GmPRR7^{H3}$	$GmPRR8^{H1}$	$GmPRR9^{H1}$	$GmPRR10^{H1}$	$GmPRR11^{H1}$	$GmPRR12^{H3}$	$GmPRR13^{H1}$	$GmPRR14^{H2}$
Tiefeng8	$GmPRR1^{H4}$	$GmPRR2^{H4}$	$GmPRR3^{H2}$	$GmPRR4^{H1}$	$GmPRR5^{H1}$	$GmPRR6^{H2}$	$GmPRR7^{H3}$	$GmPRR8^{H1}$	$GmPRR9^{H1}$	$GmPRR10^{H1}$	$GmPRR11^{H1}$	$GmPRR12^{H3}$	$GmPRR13^{H2}$	$GmPRR14^{H3}$
Fengdihuang	$GmPRR1^{H4}$	$GmPRR2^{H4}$	$GmPRR3^{H2}$	$GmPRR4^{H1}$	$GmPRR5^{H1}$	$GmPRR6^{H3}$	$GmPRR7^{H3}$	$GmPRR8^{H1}$	$GmPRR9^{H1}$	$GmPRR10^{H1}$	$GmPRR11^{H1}$	$GmPRR12^{H3}$	$GmPRR13^{H1}$	$GmPRR14^{H3}$
Changpingqingdou	$GmPRR1^{H4}$	$GmPRR2^{H4}$	$GmPRR3^{H2}$	$GmPRR4^{H1}$	$GmPRR5^{H1}$	$GmPRR6^{H3}$	$GmPRR7^{H3}$	/	$GmPRR9^{H3}$	$GmPRR10^{H1}$	/	$GmPRR12^{H3}$	$GmPRR13^{H1}$	$GmPRR14^{H2}$
Jindou25	$GmPRR1^{H4}$	$GmPRR2^{H4}$	$GmPRR3^{H2}$	$GmPRR4^{H1}$	$GmPRR5^{H1}$	$GmPRR6^{H3}$	$GmPRR7^{H4}$	$GmPRR8^{H2}$	/	$GmPRR10^{H1}$	$GmPRR11^{H2}$	$GmPRR12^{H3}$	$GmPRR13^{H2}$	$GmPRR14^{H3}$
Qiandou6	$GmPRR1^{H4}$	$GmPRR2^{H4}$	$GmPRR3^{H2}$	$GmPRR4^{H1}$	$GmPRR5^{H4}$	/	/	$GmPRR8^{H1}$	$GmPRR9^{H2}$	$GmPRR10^{H1}$	$GmPRR11^{H2}$	$GmPRR12^{H1}$	$GmPRR13^{H2}$	$GmPRR14^{H2}$
Zaofeng1	$GmPRR1^{H4}$	$GmPRR2^{H4}$	$GmPRR3^{H2}$	$GmPRR4^{H2}$	$GmPRR5^{H1}$	$GmPRR6^{H2}$	$GmPRR7^{H3}$	$GmPRR8^{H2}$	$GmPRR9^{H1}$	$GmPRR10^{H1}$	$GmPRR11^{H1}$	$GmPRR12^{H3}$	$GmPRR13^{H1}$	$GmPRR14^{H2}$
Jinjiangdaqingren	$GmPRR1^{H4}$	$GmPRR2^{H4}$	$GmPRR3^{H2}$	$GmPRR4^{H2}$	$GmPRR5^{H1}$	$GmPRR6^{H3}$	$GmPRR7^{H4}$	$GmPRR8^{H1}$	$GmPRR9^{H1}$	$GmPRR10^{H1}$	$GmPRR11^{H1}$	$GmPRR12^{H1}$	$GmPRR13^{H1}$	$GmPRR143^{2}$
Yangchunqingpidou	$GmPRR1^{H4}$	$GmPRR2^{H4}$	$GmPRR3^{H2}$	$GmPRR4^{H2}$	$GmPRR5^{H1}$	$GmPRR6^{H3}$	$GmPRR7^{H3}$	$GmPRR8^{H2}$	$GmPRR9^{H3}$	$GmPRR10^{H1}$	$GmPRR11^{H1}$	$GmPRR12^{H5}$	$GmPRR13^{H2}$	$GmPRR14^{H2}$
Bedford	$GmPRR1^{H4}$	$GmPRR2^{H4}$	$GmPRR3^{H2}$	$GmPRR4^{H2}$	$GmPRR5^{H1}$	$GmPRR6^{H4}$	$GmPRR7^{H3}$	$GmPRR8^{H1}$	$GmPRR9^{H1}$	$GmPRR10^{H1}$	$GmPRR11^{H2}$	$GmPRR12^{H1}$	$GmPRR13^{H2}$	$GmPRR14^{H2}$
Hezeniumaohuang	$GmPRR1^{H4}$	$GmPRR2^{H4}$	$GmPRR3^{H2}$	$GmPRR4^{H2}$	/	$GmPRR6^{H4}$	$GmPRR7^{H4}$	$GmPRR8^{H1}$	$GmPRR9^{H1}$	$GmPRR10^{H1}$	$GmPRR11^{H1}$	$GmPRR12^{H3}$	$GmPRR13^{H2}$	$GmPRR14^{H3}$
Xudou5	$GmPRR1^{H4}$	$GmPRR2^{H4}$	$GmPRR3^{H2}$	$GmPRR4^{H2}$	$GmPRR5^{H2}$	$GmPRR6^{H2}$	$GmPRR7^{H2}$	$GmPRR8^{H2}$	$GmPRR9^{H1}$	$GmPRR10^{H1}$	$GmPRR11^{H2}$	$GmPRR12^{H1}$	$GmPRR13^{H2}$	$GmPRR14^{H2}$
Yudou2	$GmPRR1^{H4}$	$GmPRR2^{H4}$	$GmPRR3^{H2}$	$GmPRR4^{H2}$	$GmPRR5^{H4}$	$GmPRR6^{H4}$	$GmPRR7^{H3}$	$GmPRR8^{H1}$	$GmPRR9^{H1}$	$GmPRR10^{H1}$	$GmPRR11^{H2}$	$GmPRR12^{H3}$	$GmPRR13^{H1}$	$GmPRR14^{H2}$
Xudou2	$GmPRR1^{H4}$	$GmPRR2^{H4}$	$GmPRR3^{H2}$	$GmPRR4^{H2}$	$GmPRR5^{H4}$	$GmPRR6^{H2}$	$GmPRR7^{H4}$	$GmPRR8^{H2}$	$GmPRR9^{H1}$	$GmPRR10^{H1}$	$GmPRR11^{H2}$	$GmPRR12^{H1}$	$GmPRR13^{H2}$	$GmPRR14^{H2}$
Zaofeng12	$GmPRR1^{H4}$	$GmPRR2^{H4}$	$GmPRR3^{H3}$	$GmPRR4^{H1}$	$GmPRR5^{H1}$	$GmPRR6^{H1}$	$GmPRR7^{H3}$	$GmPRR8^{H1}$	$GmPRR9^{H1}$	$GmPRR10^{H1}$	$GmPRR11^{H1}$	$GmPRR12^{H3}$	$GmPRR13^{H1}$	$GmPRR14^{H2}$
Heihe18	$GmPRR1^{H4}$	$GmPRR2^{H4}$	$GmPRR3^{H3}$	$GmPRR4^{H1}$	$GmPRR5^{H1}$	$GmPRR6^{H1}$	$GmPRR7^{H3}$	$GmPRR8^{H1}$	$GmPRR9^{H1}$	$GmPRR10^{H1}$	$GmPRR11^{H1}$	$GmPRR12^{H3}$	$GmPRR13^{H1}$	$GmPRR14^{H2}$
Kaiyu10	$GmPRR1^{H4}$	$GmPRR2^{H4}$	$GmPRR3^{H3}$	$GmPRR4^{H1}$	$GmPRR5^{H1}$	$GmPRR6^{H1}$	$GmPRR7^{H3}$	$GmPRR8^{H1}$	$GmPRR9^{H1}$	$GmPRR10^{H1}$	$GmPRR11^{H1}$	$GmPRR12^{H3}$	$GmPRR13^{H1}$	$GmPRR14^{H4}$
Portage	$GmPRR1^{H4}$	$GmPRR2^{H4}$	$GmPRR3^{H3}$	$GmPRR4^{H1}$	$GmPRR5^{H1}$	$GmPRR6^{H1}$	$GmPRR7^{H3}$	$GmPRR8^{H1}$	$GmPRR9^{H1}$	$GmPRR10^{H1}$	$GmPRR11^{H1}$	$GmPRR12^{H3}$	$GmPRR13^{H2}$	$GmPRR14^{H2}$
Dongnong72-806	$GmPRR1^{H4}$	$GmPRR2^{H4}$	$GmPRR3^{H3}$	$GmPRR4^{H1}$	$GmPRR5^{H1}$	$GmPRR6^{H1}$	$GmPRR7^{H3}$	$GmPRR8^{H1}$	$GmPRR9^{H1}$	$GmPRR10^{H1}$	$GmPRR11^{H1}$	$GmPRR12^{H3}$	$GmPRR13^{H2}$	$GmPRR14^{H2}$

**Variety**	***GmPRR1***	***GmPRR2***	***GmPRR3***	***GmPRR4***	***GmPRR5***	***GmPRR6***	***GmPRR7***	***GmPRR8***	***GmPRR9***	***GmPRR10***	***GmPRR11***	***GmPRR12***	***GmPRR13***	***GmPRR14***
Zihua4	*GmPRR1*^{H4}	*GmPRR2*^{H4}	*GmPRR3*^{H3}	*GmPRR4*^{H1}	*GmPRR5*^{H1}	*GmPRR6*^{H1}	*GmPRR7*^{H3}	*GmPRR8*^{H2}	*GmPRR9*^{H1}	*GmPRR10*^{H1}	*GmPRR11*^{H1}	*GmPRR12*^{H3}	*GmPRR13*^{H1}	*GmPRR14*^{H2}
Evans	*GmPRR1*^{H4}	*GmPRR2*^{H4}	*GmPRR3*^{H3}	*GmPRR4*^{H1}	*GmPRR5*^{H1}	*GmPRR6*^{H1}	*GmPRR7*^{H3}	*GmPRR8*^{H2}	*GmPRR9*^{H1}	*GmPRR10*^{H1}	*GmPRR11*^{H1}	*GmPRR12*^{H3}	*GmPRR13*^{H1}	*GmPRR14*^{H2}
Jiyu57	*GmPRR1*^{H4}	*GmPRR2*^{H4}	*GmPRR3*^{H3}	*GmPRR4*^{H1}	*GmPRR5*^{H1}	*GmPRR6*^{H2}	*GmPRR7*^{H2}	*GmPRR8*^{H1}	*GmPRR9*^{H1}	*GmPRR10*^{H1}	*GmPRR11*^{H1}	*GmPRR12*^{H3}	*GmPRR13*^{H1}	*GmPRR14*^{H2}
Fengshou10	*GmPRR1*^{H4}	*GmPRR2*^{H4}	*GmPRR3*^{H3}	*GmPRR4*^{H1}	*GmPRR5*^{H1}	*GmPRR6*^{H2}	*GmPRR7*^{H3}	*GmPRR8*^{H1}	*GmPRR9*^{H1}	*GmPRR10*^{H1}	*GmPRR11*^{H1}	*GmPRR12*^{H1}	*GmPRR13*^{H2}	*GmPRR14*^{H2}
Fengshou17	*GmPRR1*^{H4}	*GmPRR2*^{H4}	*GmPRR3*^{H3}	*GmPRR4*^{H1}	*GmPRR5*^{H1}	*GmPRR6*^{H2}	*GmPRR7*^{H3}	*GmPRR8*^{H1}	*GmPRR9*^{H1}	*GmPRR10*^{H1}	*GmPRR11*^{H1}	*GmPRR12*^{H1}	*GmPRR13*^{H2}	*GmPRR14*^{H2}
Liaodou15	*GmPRR1*^{H4}	*GmPRR2*^{H4}	*GmPRR3*^{H3}	*GmPRR4*^{H1}	*GmPRR5*^{H1}	*GmPRR6*^{H2}	*GmPRR7*^{H3}	*GmPRR8*^{H1}	*GmPRR9*^{H1}	*GmPRR10*^{H1}	*GmPRR11*^{H1}	*GmPRR12*^{H3}	*GmPRR13*^{H2}	*GmPRR14*^{H4}
Yunyizao	*GmPRR1*^{H4}	*GmPRR2*^{H4}	*GmPRR3*^{H3}	*GmPRR4*^{H1}	*GmPRR5*^{H1}	*GmPRR6*^{H2}	*GmPRR7*^{H4}	*GmPRR8*^{H3}	*GmPRR9*^{H2}	*GmPRR10*^{H1}	*GmPRR11*^{H2}	*GmPRR12*^{H3}	*GmPRR13*^{H2}	*GmPRR14*^{H2}
Baihuadou	*GmPRR1*^{H4}	*GmPRR2*^{H4}	*GmPRR3*^{H3}	*GmPRR4*^{H1}	/	*GmPRR6*^{H3}	*GmPRR7*^{H3}	*GmPRR8*^{H1}	*GmPRR9*^{H1}	*GmPRR10*^{H1}	*GmPRR11*^{H1}	*GmPRR12*^{H3}	*GmPRR13*^{H2}	*GmPRR14*^{H2}
Dandou2	*GmPRR1*^{H4}	*GmPRR2*^{H4}	*GmPRR3*^{H3}	*GmPRR4*^{H1}	*GmPRR5*^{H1}	*GmPRR6*^{H3}	*GmPRR7*^{H3}	*GmPRR8*^{H2}	*GmPRR9*^{H1}	*GmPRR10*^{H2}	*GmPRR11*^{H1}	*GmPRR12*^{H3}	*GmPRR13*^{H1}	*GmPRR14*^{H3}
Houzimao	*GmPRR1*^{H4}	*GmPRR2*^{H4}	*GmPRR3*^{H3}	*GmPRR4*^{H1}	*GmPRR5*^{H1}	*GmPRR6*^{H4}	*GmPRR7*^{H2}	*GmPRR8*^{H1}	*GmPRR9*^{H4}	*GmPRR10*^{H1}	*GmPRR11*^{H1}	*GmPRR12*^{H1}	*GmPRR13*^{H2}	*GmPRR14*^{H2}
Tracy	*GmPRR1*^{H4}	*GmPRR2*^{H4}	*GmPRR3*^{H3}	*GmPRR4*^{H1}	*GmPRR5*^{H1}	*GmPRR6*^{H4}	*GmPRR7*^{H3}	*GmPRR8*^{H1}	*GmPRR9*^{H4}	*GmPRR10*^{H1}	*GmPRR11*^{H1}	*GmPRR12*^{H3}	*GmPRR13*^{H2}	*GmPRR14*^{H2}
Guichun8	*GmPRR1*^{H4}	*GmPRR2*^{H4}	*GmPRR3*^{H3}	*GmPRR4*^{H1}	/	*GmPRR6*^{H4}	*GmPRR7*^{H3}	*GmPRR8*^{H3}	*GmPRR9*^{H1}	*GmPRR10*^{H1}	*GmPRR11*^{H2}	*GmPRR12*^{H1}	*GmPRR13*^{H2}	*GmPRR14*^{H2}
Merit	*GmPRR1*^{H4}	*GmPRR2*^{H4}	*GmPRR3*^{H3}	*GmPRR4*^{H1}	*GmPRR5*^{H1}	*GmPRR6*^{H1}	*GmPRR7*^{H3}	*GmPRR8*^{H2}	*GmPRR9*^{H1}	*GmPRR10*^{H1}	*GmPRR11*^{H1}	*GmPRR12*^{H2}	*GmPRR13*^{H1}	*GmPRR14*^{H3}
Taiwan75	*GmPRR1*^{H4}	*GmPRR2*^{H4}	*GmPRR3*^{H3}	*GmPRR4*^{H2}	*GmPRR5*^{H1}	*GmPRR6*^{H1}	*GmPRR7*^{H3}	*GmPRR8*^{H2}	*GmPRR9*^{H2}	*GmPRR10*^{H1}	*GmPRR11*^{H1}	*GmPRR12*^{H2}	*GmPRR13*^{H1}	*GmPRR14*^{H2}
Nandou12	*GmPRR1*^{H4}	*GmPRR2*^{H4}	*GmPRR3*^{H3}	*GmPRR4*^{H2}	*GmPRR5*^{H1}	*GmPRR6*^{H2}	*GmPRR7*^{H2}	*GmPRR8*^{H1}	*GmPRR9*^{H2}	*GmPRR10*^{H3}	*GmPRR11*^{H2}	*GmPRR12*^{H1}	*GmPRR13*^{H2}	*GmPRR14*^{H2}
Yudou8	*GmPRR1*^{H4}	*GmPRR2*^{H4}	*GmPRR3*^{H3}	*GmPRR4*^{H2}	*GmPRR5*^{H1}	*GmPRR6*^{H2}	/	*GmPRR8*^{H2}	*GmPRR9*^{H1}	*GmPRR10*^{H1}	/	*GmPRR12*^{H1}	*GmPRR13*^{H2}	*GmPRR14*^{H2}
Xiangdou3	*GmPRR1*^{H4}	*GmPRR2*^{H4}	*GmPRR3*^{H3}	*GmPRR4*^{H2}	*GmPRR5*^{H1}	*GmPRR6*^{H3}	*GmPRR7*^{H3}	*GmPRR8*^{H1}	*GmPRR9*^{H3}	*GmPRR10*^{H1}	*GmPRR11*^{H1}	*GmPRR12*^{H3}	*GmPRR13*^{H1}	*GmPRR14*^{H2}
Dandou1	*GmPRR1*^{H4}	*GmPRR2*^{H4}	*GmPRR3*^{H3}	*GmPRR4*^{H2}	*GmPRR5*^{H1}	*GmPRR6*^{H4}	*GmPRR7*^{H3}	*GmPRR8*^{H1}	*GmPRR9*^{H2}	*GmPRR10*^{H1}	*GmPRR11*^{H1}	*GmPRR12*^{H3}	*GmPRR13*^{H2}	*GmPRR14*^{H2}
77-14	*GmPRR1*^{H4}	*GmPRR2*^{H4}	*GmPRR3*^{H3}	*GmPRR4*^{H2}	*GmPRR5*^{H1}	*GmPRR6*^{H4}	*GmPRR7*^{H3}	*GmPRR8*^{H1}	*GmPRR9*^{H3}	*GmPRR10*^{H2}	*GmPRR11*^{H1}	*GmPRR12*^{H3}	*GmPRR13*^{H2}	*GmPRR14*^{H2}
Lanxidaqingdou	*GmPRR1*^{H4}	*GmPRR2*^{H4}	*GmPRR3*^{H3}	*GmPRR4*^{H2}	*GmPRR5*^{H1}	*GmPRR6*^{H4}	*GmPRR7*^{H3}	*GmPRR8*^{H2}	*GmPRR9*^{H3}	*GmPRR10*^{H1}	*GmPRR11*^{H1}	*GmPRR12*^{H3}	*GmPRR13*^{H2}	*GmPRR14*^{H2}
Yulindahuangdou	*GmPRR1*^{H4}	*GmPRR2*^{H4}	*GmPRR3*^{H3}	*GmPRR4*^{H2}	*GmPRR5*^{H1}	*GmPRR6*^{H4}	*GmPRR7*^{H3}	*GmPRR8*^{H2}	*GmPRR9*^{H4}	*GmPRR10*^{H1}	*GmPRR11*^{H2}	*GmPRR12*^{H4}	*GmPRR13*^{H2}	*GmPRR14*^{H2}
Qiudou1	*GmPRR1*^{H4}	*GmPRR2*^{H4}	*GmPRR3*^{H3}	*GmPRR4*^{H2}	*GmPRR5*^{H1}	*GmPRR6*^{H5}	*GmPRR7*^{H2}	/	*GmPRR9*^{H3}	*GmPRR10*^{H3}	*GmPRR11*^{H2}	/	*GmPRR13*^{H2}	/
Heibiqing	*GmPRR1*^{H4}	*GmPRR2*^{H4}	*GmPRR3*^{H3}	*GmPRR4*^{H2}	*GmPRR5*^{H3}	*GmPRR6*^{H3}	*GmPRR7*^{H4}	*GmPRR8*^{H2}	*GmPRR9*^{H1}	*GmPRR10*^{H3}	*GmPRR11*^{H1}	*GmPRR12*^{H3}	*GmPRR13*^{H2}	*GmPRR14*^{H2}
Liuyuehuang	*GmPRR1*^{H4}	*GmPRR2*^{H4}	*GmPRR3*^{H3}	*GmPRR4*^{H2}	*GmPRR5*^{H3}	*GmPRR6*^{H3}	*GmPRR7*^{H3}	*GmPRR8*^{H2}	*GmPRR9*^{H4}	*GmPRR10*^{H1}	*GmPRR11*^{H2}	*GmPRR12*^{H3}	*GmPRR13*^{H2}	*GmPRR14*^{H2}

Variety	*GmPRR1*	*GmPRR2*	*GmPRR3*	*GmPRR4*	*GmPRR5*	*GmPRR6*	*GmPRR7*	*GmPRR8*	*GmPRR9*	*GmPRR10*	*GmPRR11*	*GmPRR12*	*GmPRR13*	*GmPRR14*
Yuejin5	*GmPRR1*^{H4}	*GmPRR2*^{H4}	*GmPRR3*^{H3}	*GmPRR4*^{H2}	*GmPRR5*^{H4}	*GmPRR6*^{H1}	*GmPRR7*^{H3}	*GmPRR8*^{H1}	*GmPRR9*^{H1}	*GmPRR10*^{H1}	*GmPRR11*^{H2}	*GmPRR12*^{H3}	*GmPRR13*^{H2}	*GmPRR14*^{H2}
Jidou17	*GmPRR1*^{H4}	*GmPRR2*^{H4}	*GmPRR3*^{H3}	*GmPRR4*^{H2}	*GmPRR5*^{H4}	*GmPRR6*^{H2}	*GmPRR7*^{H3}	*GmPRR8*^{H2}	*GmPRR9*^{H1}	*GmPRR10*^{H1}	*GmPRR11*^{H1}	*GmPRR12*^{H1}	*GmPRR13*^{H2}	*GmPRR14*^{H2}
Youbian30	*GmPRR1*^{H4}	*GmPRR2*^{H4}	*GmPRR3*^{H3}	*GmPRR4*^{H2}	*GmPRR5*^{H4}	*GmPRR6*^{H4}	*GmPRR7*^{H2}	*GmPRR8*^{H2}	*GmPRR9*^{H1}	*GmPRR10*^{H1}	*GmPRR11*^{H2}	*GmPRR12*^{H1}	*GmPRR13*^{H2}	*GmPRR14*^{H2}
Guichun1	*GmPRR1*^{H4}	*GmPRR2*^{H4}	*GmPRR3*^{H3}	*GmPRR4*^{H2}	*GmPRR5*^{H4}	*GmPRR6*^{H4}	/	*GmPRR8*^{H3}	*GmPRR9*^{H1}	*GmPRR10*^{H1}	*GmPRR11*^{H2}	*GmPRR12*^{H1}	*GmPRR13*^{H2}	*GmPRR14*^{H2}
Jinningdahuangdou	*GmPRR1*^{H4}	*GmPRR2*^{H4}	*GmPRR3*^{H3}	*GmPRR4*^{H2}	*GmPRR5*^{H4}	*GmPRR6*^{H4}	*GmPRR7*^{H3}	*GmPRR8*^{H1}	*GmPRR9*^{H3}	*GmPRR10*^{H1}	*GmPRR11*^{H1}	*GmPRR12*^{H3}	*GmPRR13*^{H2}	/
Jupiter	*GmPRR1*^{H4}	*GmPRR2*^{H4}	*GmPRR3*^{H3}	*GmPRR4*^{H2}	*GmPRR5*^{H4}	*GmPRR6*^{H4}	*GmPRR7*^{H3}	*GmPRR8*^{H1}	*GmPRR9*^{H4}	*GmPRR10*^{H3}	*GmPRR11*^{H1}	*GmPRR12*^{H3}	*GmPRR13*^{H2}	*GmPRR14*^{H2}
Zhonghuang13	*GmPRR1*^{H4}	*GmPRR2*^{H4}	*GmPRR3*^{H3}	*GmPRR4*^{H2}	*GmPRR5*^{H4}	*GmPRR6*^{H4}	*GmPRR7*^{H3}	*GmPRR8*^{H3}	*GmPRR9*^{H1}	*GmPRR10*^{H1}	*GmPRR11*^{H1}	*GmPRR12*^{H4}	*GmPRR13*^{H2}	*GmPRR14*^{H2}
Hedou13	*GmPRR1*^{H4}	*GmPRR2*^{H4}	*GmPRR3*^{H3}	*GmPRR4*^{H2}	*GmPRR5*^{H4}	*GmPRR6*^{H4}	*GmPRR7*^{H3}	*GmPRR8*^{H3}	*GmPRR9*^{H1}	*GmPRR10*^{H1}	*GmPRR11*^{H1}	*GmPRR12*^{H4}	*GmPRR13*^{H2}	*GmPRR14*^{H2}
Zigongdongdou	*GmPRR1*^{H4}	*GmPRR2*^{H4}	*GmPRR3*^{H3}	*GmPRR4*^{H2}	*GmPRR5*^{H4}	6-2	*GmPRR7*^{H3}	*GmPRR8*^{H3}	*GmPRR9*^{H3}	*GmPRR10*^{H2}	*GmPRR11*^{H2}	*GmPRR12*^{H3}	*GmPRR13*^{H2}	*GmPRR14*^{H3}
Juhuangdadou	*GmPRR1*^{H4}	*GmPRR2*^{H4}	*GmPRR3*^{H4}	*GmPRR4*^{H2}	*GmPRR5*^{H4}	*GmPRR6*^{H4}	*GmPRR7*^{H3}	*GmPRR8*^{H3}	*GmPRR9*^{H4}	*GmPRR10*^{H1}	*GmPRR11*^{H2}	*GmPRR12*^{H4}	*GmPRR13*^{H2}	*GmPRR14*^{H2}
Wuhuasiyuehuang	*GmPRR1*^{H4}	*GmPRR2*^{H4}	*GmPRR3*^{H4}	*GmPRR4*^{H2}	*GmPRR5*^{H4}	*GmPRR6*^{H4}	*GmPRR7*^{H3}	*GmPRR8*^{H3}	*GmPRR9*^{H4}	*GmPRR10*^{H1}	*GmPRR11*^{H2}	*GmPRR12*^{H4}	*GmPRR13*^{H2}	*GmPRR14*^{H2}
Daoshuihuang	*GmPRR1*^{H4}	*GmPRR2*^{H4}	*GmPRR3*^{H4}	*GmPRR4*^{H2}	*GmPRR5*^{H4}	*GmPRR6*^{H4}	*GmPRR7*^{H3}	*GmPRR8*^{H3}	*GmPRR9*^{H4}	*GmPRR10*^{H1}	*GmPRR11*^{H2}	*GmPRR12*^{H4}	*GmPRR13*^{H2}	*GmPRR14*^{H2}
Shangraodaqingsi	*GmPRR1*^{H4}	*GmPRR2*^{H4}	*GmPRR3*^{H5}	*GmPRR4*^{H1}	*GmPRR5*^{H4}	*GmPRR6*^{H5}	7-2	*GmPRR8*^{H2}	*GmPRR9*^{H3}	*GmPRR10*^{H3}	*GmPRR11*^{H2}	*GmPRR12*^{H3}	*GmPRR13*^{H2}	*GmPRR14*^{H2}
Baishuidou	*GmPRR1*^{H4}	*GmPRR2*^{H4}	/	/	*GmPRR5*^{H1}	/	/	/	/	/	*GmPRR11*^{H2}	*GmPRR12*^{H3}	*GmPRR13*^{H2}	*GmPRR14*^{H2}
Beeson	*GmPRR1*^{H4}	*GmPRR2*^{H5}	*GmPRR3*^{H1}	*GmPRR4*^{H1}	*GmPRR5*^{H1}	*GmPRR6*^{H1}	*GmPRR7*^{H3}	*GmPRR8*^{H2}	*GmPRR9*^{H1}	*GmPRR10*^{H1}	*GmPRR11*^{H1}	*GmPRR12*^{H3}	*GmPRR13*^{H1}	*GmPRR14*^{H2}
Jindou19	*GmPRR1*^{H4}	*GmPRR2*^{H5}	*GmPRR3*^{H2}	*GmPRR4*^{H1}	*GmPRR5*^{H1}	*GmPRR6*^{H3}	*GmPRR7*^{H3}	*GmPRR8*^{H1}	*GmPRR9*^{H1}	*GmPRR10*^{H1}	*GmPRR11*^{H2}	*GmPRR12*^{H3}	*GmPRR13*^{H1}	*GmPRR14*^{H3}
Tiefeng31	*GmPRR1*^{H4}	*GmPRR2*^{H5}	*GmPRR3*^{H3}	*GmPRR4*^{H1}	*GmPRR5*^{H1}	*GmPRR6*^{H2}	*GmPRR7*^{H3}	*GmPRR8*^{H1}	*GmPRR9*^{H1}	*GmPRR10*^{H1}	*GmPRR11*^{H1}	*GmPRR12*^{H2}	*GmPRR13*^{H1}	*GmPRR14*^{H2}
Centennial	*GmPRR1*^{H4}	*GmPRR2*^{H5}	*GmPRR3*^{H3}	*GmPRR4*^{H2}	*GmPRR5*^{H1}	*GmPRR6*^{H4}	*GmPRR7*^{H3}	*GmPRR8*^{H1}	*GmPRR9*^{H4}	*GmPRR10*^{H1}	*GmPRR11*^{H1}	*GmPRR12*^{H1}	*GmPRR13*^{H2}	*GmPRR14*^{H2}
Wenfeng7	*GmPRR1*^{H4}	*GmPRR2*^{H6}	*GmPRR3*^{H2}	*GmPRR4*^{H1}	*GmPRR5*^{H1}	*GmPRR6*^{H2}	*GmPRR7*^{H4}	*GmPRR8*^{H3}	*GmPRR9*^{H1}	*GmPRR10*^{H1}	*GmPRR11*^{H2}	*GmPRR12*^{H1}	*GmPRR13*^{H2}	*GmPRR14*^{H2}
Jindou3	*GmPRR1*^{H4}	*GmPRR2*^{H6}	*GmPRR3*^{H2}	*GmPRR4*^{H1}	*GmPRR5*^{H1}	*GmPRR6*^{H2}	*GmPRR7*^{H3}	*GmPRR8*^{H3}	*GmPRR9*^{H3}	*GmPRR10*^{H1}	*GmPRR11*^{H1}	*GmPRR12*^{H3}	*GmPRR13*^{H1}	*GmPRR14*^{H3}
Naiyinheidou	*GmPRR1*^{H4}	*GmPRR2*^{H6}	*GmPRR3*^{H2}	*GmPRR4*^{H1}	*GmPRR5*^{H4}	*GmPRR6*^{H2}	*GmPRR7*^{H4}	*GmPRR8*^{H3}	*GmPRR9*^{H1}	*GmPRR10*^{H3}	*GmPRR11*^{H2}	*GmPRR12*^{H1}	*GmPRR13*^{H2}	*GmPRR14*^{H3}
Chenliuniumaohuang	*GmPRR1*^{H4}	*GmPRR2*^{H6}	*GmPRR3*^{H2}	*GmPRR4*^{H2}	*GmPRR5*^{H4}	*GmPRR6*^{H4}	*GmPRR7*^{H4}	*GmPRR8*^{H1}	*GmPRR9*^{H4}	*GmPRR10*^{H1}	*GmPRR11*^{H1}	*GmPRR12*^{H3}	*GmPRR13*^{H2}	*GmPRR14*^{H2}
Bahong1	*GmPRR1*^{H4}	*GmPRR2*^{H6}	*GmPRR3*^{H3}	*GmPRR4*^{H1}	*GmPRR5*^{H1}	*GmPRR6*^{H3}	*GmPRR7*^{H4}	*GmPRR8*^{H3}	*GmPRR9*^{H1}	/	*GmPRR11*^{H2}	*GmPRR12*^{H3}	*GmPRR13*^{H2}	*GmPRR14*^{H5}
Hengyangbayueqing	*GmPRR1*^{H4}	*GmPRR2*^{H6}	*GmPRR3*^{H3}	*GmPRR4*^{H1}	*GmPRR5*^{H1}	*GmPRR6*^{H4}	*GmPRR7*^{H2}	*GmPRR8*^{H1}	*GmPRR9*^{H3}	*GmPRR10*^{H1}	*GmPRR11*^{H1}	*GmPRR12*^{H3}	*GmPRR13*^{H2}	*GmPRR14*^{H2}

**Variety**	***GmPRR1***	***GmPRR2***	***GmPRR3***	***GmPRR4***	***GmPRR5***	***GmPRR6***	***GmPRR7***	***GmPRR8***	***GmPRR9***	***GmPRR10***	***GmPRR11***	***GmPRR12***	***GmPRR13***	***GmPRR14***
Miquanhuangdou	*GmPRR1*^{H4}	*GmPRR2*^{H6}	*GmPRR3*^{H3}	*GmPRR4*^{H2}	*GmPRR5*^{H1}	*GmPRR6*^{H1}	*GmPRR7*^{H1}	*GmPRR8*^{H3}	*GmPRR9*^{H1}	*GmPRR10*^{H1}	*GmPRR11*^{H1}	*GmPRR12*^{H1}	*GmPRR13*^{H2}	*GmPRR14*^{H3}
Yanhuang1	*GmPRR1*^{H4}	*GmPRR2*^{H6}	*GmPRR3*^{H3}	*GmPRR4*^{H2}	*GmPRR5*^{H1}	*GmPRR6*^{H4}	*GmPRR7*^{H3}	*GmPRR8*^{H3}	/	*GmPRR10*^{H1}	*GmPRR11*^{H2}	*GmPRR12*^{H3}	*GmPRR13*^{H3}	*GmPRR14*^{H2}
Ludou4	*GmPRR1*^{H4}	*GmPRR2*^{H6}	*GmPRR3*^{H3}	*GmPRR4*^{H2}	*GmPRR5*^{H1}	*GmPRR6*^{H4}	*GmPRR7*^{H3}	/	*GmPRR9*^{H1}	*GmPRR10*^{H1}	*GmPRR11*^{H2}	*GmPRR12*^{H4}	/	*GmPRR14*^{H2}
Weiqingdou	*GmPRR1*^{H4}	*GmPRR2*^{H6}	*GmPRR3*^{H3}	*GmPRR4*^{H2}	*GmPRR5*^{H1}	*GmPRR6*^{H4}	*GmPRR7*^{H3}	*GmPRR8*^{H1}	*GmPRR9*^{H1}	*GmPRR10*^{H1}	*GmPRR11*^{H1}	*GmPRR12*^{H3}	*GmPRR13*^{H2}	*GmPRR14*^{H2}
Yuejin4	*GmPRR1*^{H4}	*GmPRR2*^{H6}	*GmPRR3*^{H3}	*GmPRR4*^{H2}	*GmPRR5*^{H1}	*GmPRR6*^{H4}	*GmPRR7*^{H4}	*GmPRR8*^{H3}	*GmPRR9*^{H1}	*GmPRR10*^{H1}	*GmPRR11*^{H2}	*GmPRR12*^{H3}	*GmPRR13*^{H3}	*GmPRR14*^{H2}
Heinong48	*GmPRR1*^{H4}	/	*GmPRR3*^{H1}	*GmPRR4*^{H1}	*GmPRR5*^{H1}	*GmPRR6*^{H2}	/	*GmPRR8*^{H1}	*GmPRR9*^{H1}	*GmPRR10*^{H1}	*GmPRR11*^{H1}	*GmPRR12*^{H1}	*GmPRR13*^{H2}	*GmPRR14*^{H2}
Jiti5	*GmPRR1*^{H4}	/	*GmPRR3*^{H2}	*GmPRR4*^{H1}	*GmPRR5*^{H1}	*GmPRR6*^{H1}	*GmPRR7*^{H3}	*GmPRR8*^{H1}	*GmPRR9*^{H1}	*GmPRR10*^{H1}	*GmPRR11*^{H1}	*GmPRR12*^{H3}	*GmPRR13*^{H1}	*GmPRR14*^{H2}
Ludou11	*GmPRR1*^{H4}	/	*GmPRR3*^{H2}	*GmPRR4*^{H1}	*GmPRR5*^{H1}	*GmPRR6*^{H4}	*GmPRR7*^{H3}	/	*GmPRR9*^{H1}	*GmPRR10*^{H1}	*GmPRR11*^{H2}	*GmPRR12*^{H3}	*GmPRR13*^{H1}	*GmPRR14*^{H2}
Dian86-4	*GmPRR1*^{H4}	/	*GmPRR3*^{H1}	/	*GmPRR5*^{H1}	*GmPRR6*^{H4}	*GmPRR7*^{H3}	*GmPRR8*^{H1}	*GmPRR9*^{H3}	*GmPRR10*^{H1}	*GmPRR11*^{H1}	*GmPRR12*^{H3}	*GmPRR13*^{H2}	*GmPRR14*^{H3}
PI 548655	*GmPRR1*^{H5}	*GmPRR2*^{H4}	*GmPRR3*^{H2}	*GmPRR4*^{H2}	*GmPRR5*^{H1}	*GmPRR6*^{H4}	*GmPRR7*^{H3}	*GmPRR8*^{H1}	*GmPRR9*^{H1}	*GmPRR10*^{H1}	*GmPRR11*^{H2}	*GmPRR12*^{H3}	*GmPRR13*^{H2}	*GmPRR14*^{H2}
PI 548660	*GmPRR1*^{H5}	*GmPRR2*^{H4}	*GmPRR3*^{H3}	*GmPRR4*^{H2}	*GmPRR5*^{H1}	*GmPRR6*^{H4}	*GmPRR7*^{H3}	*GmPRR8*^{H1}	*GmPRR9*^{H4}	*GmPRR10*^{H1}	*GmPRR11*^{H2}	*GmPRR12*^{H1}	*GmPRR13*^{H2}	*GmPRR14*^{H2}
Braxton	*GmPRR1*^{H5}	*GmPRR2*^{H4}	*GmPRR3*^{H3}	*GmPRR4*^{H2}	*GmPRR5*^{H1}	*GmPRR6*^{H4}	*GmPRR7*^{H3}	*GmPRR8*^{H1}	*GmPRR9*^{H4}	*GmPRR10*^{H1}	*GmPRR11*^{H2}	*GmPRR12*^{H1}	*GmPRR13*^{H2}	*GmPRR14*^{H2}
Cutler	*GmPRR1*^{H5}	*GmPRR2*^{H5}	*GmPRR3*^{H2}	*GmPRR4*^{H1}	*GmPRR5*^{H1}	*GmPRR6*^{H2}	*GmPRR7*^{H3}	*GmPRR8*^{H1}	*GmPRR9*^{H1}	*GmPRR10*^{H1}	*GmPRR11*^{H1}	*GmPRR12*^{H1}	*GmPRR13*^{H1}	*GmPRR14*^{H2}
Dare	*GmPRR1*^{H5}	*GmPRR2*^{H5}	*GmPRR3*^{H2}	*GmPRR4*^{H2}	*GmPRR5*^{H1}	*GmPRR6*^{H1}	*GmPRR7*^{H3}	*GmPRR8*^{H1}	*GmPRR9*^{H1}	*GmPRR10*^{H1}	*GmPRR11*^{H1}	*GmPRR12*^{H3}	*GmPRR13*^{H2}	*GmPRR14*^{H4}
Hood	*GmPRR1*^{H5}	*GmPRR2*^{H5}	*GmPRR3*^{H3}	*GmPRR4*^{H1}	*GmPRR5*^{H1}	*GmPRR6*^{H1}	*GmPRR7*^{H3}	*GmPRR8*^{H2}	*GmPRR9*^{H1}	*GmPRR10*^{H1}	*GmPRR11*^{H2}	*GmPRR12*^{H1}	*GmPRR13*^{H1}	*GmPRR14*^{H2}
PI 548663	*GmPRR1*^{H5}	*GmPRR2*^{H5}	*GmPRR3*^{H3}	*GmPRR4*^{H2}	*GmPRR5*^{H1}	*GmPRR6*^{H4}	*GmPRR7*^{H3}	*GmPRR8*^{H1}	*GmPRR9*^{H4}	*GmPRR10*^{H1}	*GmPRR11*^{H1}	*GmPRR12*^{H1}	*GmPRR13*^{H2}	*GmPRR14*^{H2}

* Each line in the table represents the division of different haplotype combinations. ″/″ represents that the loci in *GmPRRs* of the variety has not been detected.

Supplementary Table S4. Primer sequences used for qRT-PCR analysis of *GmPRRs*

Gene name	Forward primer sequence (5′-3′)	Reverse primer sequence (5′-3′)
GmPRR1	ACAAGGGTTCGATGCAGACC	TTCGACAAGCAAGACCCGAA
GmPRR2	AGCAACTTGGAAGGGGTTGT	ATGCCTCTCCATTTTCCCGT
GmPRR3	TGACCCTGTCGGTAACATGC	CGTGCTTGCCTCACCATTTT
GmPRR4	GCGCATGCTTGGACTTGTAG	CAGCAGCAATGGTGGCAATA
GmPRR5	CAGCTTCAGAGGGCAAGCAT	GGATGCACTTGACGCACAAA
GmPRR6	GAATGTGGGCCTGATGTGGA	ACTTCCATCCCAACATGCGT
GmPRR7	ATCCAAGAACGCCCCAGATG	GCCAGTCTTTTCCGGCTTTG
GmPRR8	GCAACAATTCTGGCACCACC	GTGGAGCAGCAGTAGCCATT
GmPRR9	AAGTTGGCAGTCCTCATCCG	GTCGCATGCACCCATCTACT
GmPRR10	GAAAGTGCGACCCTCACCAG	TCTGAGTGCCACTTCCATTGT
GmPRR11	CACAGCCTCAAGGGGCAATA	ACAATTGCGAAGCAGTGCAG
GmPRR12	TGCATGGATGCAATGCTGAG	GCTTGTTTACCTGAGCTGCC
GmPRR13	GTCAACAGAAAACGGCTCGC	AGTGGAAGCAGGTTGTCCAT
GmPRR14	TGGAGAAGGCACGCCATAAG	CCAAAAATGGCGATGGGCAA
GmActin	CGGTGGTTCTATCTTGGCATC	GTCTTTCGCTTCAATAACCCTA
